# Smoking Amplifies Comorbidity-Associated Risk in Orthopaedic Surgery: A Multiplicative Interaction

**DOI:** 10.3390/jcm14228217

**Published:** 2025-11-19

**Authors:** Edith Simona Ianoși, Daria-Maria Roșu, Arpad Solyom, Bianca Liana Grigorescu, Mara Vultur, Maria Beatrice Ianoși

**Affiliations:** 1 Department of Pulmonology, University of Medicine, Pharmacy, Technology and Sciences “George Emil Palade” of Târgu Mureș, 540139 Târgu Mures, Romania; edith.ianosi@umfst.ro (E.S.I.);; 2 Faculty of General Medicine, University of Medicine, Pharmacy, Science and Technology “George Emil Palade” of Târgu Mureș, 540139 Târgu Mures, Romania; 3 Department of Orthopaedic and Traumatology Surgery, University of Medicine, Pharmacy, Science and Technology “George Emil Palade” of Târgu Mureș, 540139 Târgu Mures, Romania; 4 Department of Anaesthesiology and Intensive Care, University of Medicine, Pharmacy, Science and Technology “George Emil Palade” of Târgu Mureș, 540139 Târgu Mures, Romania; 5 Pulmonology Clinic, Mures, County Clinical Hospital, 540011 Târgu Mures, Romania

**Keywords:** tobacco use, postoperative complications, orthopaedic procedures, diabetes mellitus, anemia, venous insufficiency, hepatitis, tuberculosis, COPD, risk assessment

## Abstract

**Background:** The success of orthopaedic surgery is fundamentally biological, yet the synergistic effect of smoking and comorbidities on surgical outcomes is not well quantified. We hypothesised that active smoking multiplies the risk conferred by common comorbidities. **Methods:** In this retrospective cohort study, we analysed 3123 orthopaedic procedures from 2020 to 2024. Patients were stratified by comorbidity (diabetes, anemia, hepatic dysfunction, chronic venous disease) and smoking status. Primary outcomes were orthopaedic-specific complications, including non-union, periprosthetic joint infection (PJI), and revision surgery. We used multivariate logistic regression to calculate adjusted odds ratios (aORs) and formal tests for interaction to quantify synergy. **Results:** A significant synergistic effect was observed. In analyses adjusted for age, sex, and procedure acuity, diabetic smokers had significantly higher rates of non-union (8.6% vs. 3.3%; aOR 3.0, 95% CI 1.1–8.2), periprosthetic joint infection (8.2% vs. 2.8%; aOR 3.1, 95% CI 1.1–8.9), and revision surgery (12.2% vs. 5.0%; aOR 2.7, 95% CI 1.2–6.1). Significant interaction effects confirmed this synergy. Smokers with hepatic dysfunction had higher haematoma rates, while smoking with severe anemia was associated with markedly increased mortality (5.0%; aOR 8.9, 95% CI 1.8–43.1). Former smokers’ outcomes were consistently intermediate between active and non-smokers. For example, in the diabetic cohort, the adjusted odds of non-union were elevated for both former (aOR 2.1, 95% CI 0.8–5.5) and active smokers (aOR 3.0, 95% CI 1.1–8.2) compared to non-smokers (reference), demonstrating a gradient of risk. **Conclusions:** Smoking is associated with a multiplicative increase in comorbidity risk, creating a distinct high-risk phenotype that severely compromises healing. These findings strongly support that verified smoking cessation should be a foundational component of preoperative optimisation before elective orthopaedic surgery.

## 1. Introduction

The success of orthopaedic surgery is profoundly dependent on the body’s innate capacity for biological repair. While technical precision is crucial, a successful outcome is ultimately determined by the patient’s physiological ability to achieve fracture union, secure implant osseointegration, and mount an effective immune defence against infection—processes that are particularly vulnerable to systemic physiological compromise [1,2].

Comorbidities such as diabetes mellitus (DM), anemia, and hepatic dysfunction are established independent risk factors that erode this biological foundation [3,4,5,6]. Concurrently, cigarette smoking remains an extensive global health epidemic and a potent modifiable risk factor for surgical complications [7,8]. Its pathophysiological insults—systemic vasoconstriction, tissue hypoxia, carbon monoxide-mediated impairment of oxygen carriage, increased platelet aggregability, and direct suppression of immune cell function—may directly undermine the very processes upon which orthopaedic surgery relies [9,10,11].

While the independent risks of smoking [7,8] and comorbidities like diabetes and anemia [3,4] are well-documented, their *synergistic* effect in the context of orthopaedic surgery remains underexplored and poorly quantified. Prior studies and reviews have often evaluated these factors in isolation [12,13], creating a critical gap in understanding whether their combined effect is merely additive or potentially multiplicative. This gap is critical; the combination may result in more than additive risk, potentially creating a distinct high-risk phenotype vulnerable to catastrophic orthopaedic-specific complications like non-union, periprosthetic joint infection (PJI), and early implant failure. We hypothesized that active smoking would interact synergistically with common comorbidities to significantly increase the risk of orthopaedic-specific complications beyond their independent effects. This large, five-year retrospective cohort analysis aims to definitively characterise this synergy.

This large, five-year retrospective cohort analysis aims to definitively characterise this synergy. We hypothesise that active smoking interacts synergistically with a spectrum of comorbidities to significantly worsen preoperative physiological readiness, intraoperative stability, and, most importantly, postoperative orthopaedic-specific outcomes. By analysing over 3000 procedures, we sought to provide a comprehensive quantification of this risk and propose a paradigm shift towards integrated, comorbidity-specific perioperative optimisation pathways. With an estimated 1.1 billion smokers worldwide [8] and the rising prevalence of comorbidities like diabetes and obesity [14,15], quantifying this synergistic risk is a pressing public health priority with direct implications for global orthopaedic surgical safety.

## 2. Methods

### 2.1. Study Design and Population

We conducted a retrospective cohort study at a single high-volume tertiary academic center. To construct a representative cohort, we identified all orthopaedic surgical procedures performed during a selection of months between 1 January 2020, and 31 December 2024. A randomized selection of 4–5 months per year across the study period (2020–2024) was performed. This resulted in a total of 22 sampled months. This strategy was designed to minimize potential seasonal selection bias. The specific months were chosen using a randomized approach to mitigate seasonal bias (detailed in Supplementary Appendix Text S1).

The initial data pull identified all adult patients (≥18 years) undergoing orthopaedic procedures during the sampled months. Patients were excluded if data on smoking status or key outcome variables were missing. The number of pediatric patients (<18 years) identified in the sampled months was negligible (n < 5) and therefore did not constitute a meaningful exclusion category. After exclusions, the final master cohort for analysis consisted of 3123 unique patients (representing 3123 procedures).

It is important to note that patients with other common comorbidities (e.g., hypertension, obesity, chronic kidney disease) were not excluded from the master cohort but were not selected for the subsequent comorbidity-specific sub-cohort analysis. This approach was chosen to isolate the synergistic effect of smoking with each specific condition under study.

### 2.2. Patient Stratification and Variables

Within each comorbidity cohort, patients were stratified by smoking status based on the documentation available in the original, handwritten clinical records:

- Active Smoker: Any documented note of current tobacco use. Data on smoking intensity (e.g., cigarettes per day, pack-years) were not consistently available in the clinical records and were therefore not included in this analysis. A 6-month cutoff was not applied, as this level of temporal detail was not consistently recorded. The classification relied on the clinician’s global assessment (e.g., “smoker,” “active smoker”).
- Former Smoker: A documented history of smoking but note of abstinence (e.g., “former smoker,” “quit smoking”). Specific duration of abstinence or pack-year history were not available.
- Non-Smoker: No documented history of tobacco use.

A documented history of smoking but note of abstinence (e.g., “former smoker,” “quit smoking”). Specific duration of abstinence or pack-year history were not available.

### 2.3. Comorbidity Definitions Were Rigorously Applied

- Diabetes Mellitus (DM): n = 365; documented diagnosis or use of hypoglycaemic agents.
- Anaemia: n = 374; defined as a preoperative Hb level of <12 g/dL in women or <13 g/dL in men. A preoperative hemoglobin level was part of the standard institutional protocol for surgical clearance. The ‘available data’ refers to the results of this routinely performed test, which was available for the vast majority of patients. Patients were included in the anemia cohort if their recorded pre-op Hb met the criteria.
- Hepatic Dysfunction: n = 238; documented diagnosis of hepatitis, cirrhosis, or steatosis; or ALT/AST >1.5 × upper limit of normal.
- Chronic Venous Disease (CVD): n = 592; documented chronic venous insufficiency, varicose veins, or history of venous ulceration.
- COPD: n = 54; documented diagnosis [16].
- History of TB: n = 22; documented history of treated tuberculosis.

The six specific comorbidities (Diabetes, Anemia, Hepatic Dysfunction, Chronic Venous Disease, COPD, and Tuberculosis) were selected for this analysis based on their strong pathophysiological plausibility for interacting with smoking to impair bone healing, immune function, and perioperative stability [9,10,17,18,19,20]. While tuberculosis (TB) history was less prevalent in our cohort (n = 22), it was included due to its profound and lasting impact on systemic immune function, representing a distinct high-risk phenotype worthy of investigation [21]. Preoperative laboratory values were those recorded closest to the surgery date, typically upon hospital admission for the procedure.

For the purpose of this interaction analysis, patients were categorized into mutually exclusive comorbidity sub-cohorts. If a patient had more than one of the six index comorbidities, they were assigned to a single, pre-specified cohort based on a hierarchy of pathophysiological relevance to bone healing and infection risk (the hierarchy was: Tuberculosis > COPD > Hepatic Dysfunction > Diabetes Mellitus > Anemia > Chronic Venous Disease). This strategy was implemented to ensure statistical independence of the sub-cohorts and to allow for a clear interpretation of the interaction between smoking and each specific comorbidity.

### 2.4. Orthopaedic Surgical Procedures

To this end, we included a representative spectrum of common major orthopedic procedures that are critically dependent on successful bone union, osseointegration, and immune competence. While these procedures differ in their technical execution, they share a common fundamental reliance on the host’s physiological healing capacity, which is the primary target of smoking’s pathophysiological effects. The included procedures were as follows:

- Trauma and Fracture Fixation: Intramedullary nailing, open reduction and internal fixation (ORIF) of fractures (e.g., proximal femur, tibia).
- Arthroplasty: Primary total hip, knee, and shoulder arthroplasty.
- Spinal Surgery: Instrumented fusion.

### 2.5. Outcome Measures

Outcomes were assessed across three domains:

1. Preoperative Readiness: Hb, platelet count, INR, albumin, CRP, ASA score.
2. Intraoperative Outcomes: Estimated blood loss, transfusion requirement, and a composite “hostile field” outcome. This composite was defined pragmatically for this study as the occurrence of ≥2 of the following: estimated blood loss EBL >95th percentile for the procedure type, intraoperative transfusion, or a qualitative surgeon note of “friable tissues,” “poor bone quality,” or “persistent oozing” as manually extracted from the operative reports.
3. Postoperative Orthopaedic-Specific Outcomes (Primary): The incidence of Periprosthetic Joint Infection (PJI) as primary outcome, diagnosis based on clinical and laboratory criteria per surgeon note and diagnosed according to the 2018 International Consensus Meeting (ICM) criteria [22] as documented in the clinical record. Different outcomes were assessed based on data available from the primary surgical hospitalization and any subsequent inpatient readmissions to our institution. Follow-up data from outpatient clinic visits or non-surgical readmissions were not systematically captured in this retrospective analysis. Therefore, the reported complication rates likely represent a minimum estimate, particularly for later-presenting outcomes. They included Non-union (defined as a lack of radiographic bridging at a minimum of 6 months postoperatively, as described in radiology reports), implant failure, reoperation/revision surgery, and 30-day mortality. The lack of a standardized, long-term follow-up protocol is a limitation, and outcomes are based on the available clinical documentation. Estimated blood loss (EBL), transfusion requirement, and a composite “hostile surgical field” outcome. This composite was defined as the occurrence of two or more of the following objectively measurable or clearly documented events: (1) EBL greater than the 95th percentile for the specific procedure type, (2) requirement for an intraoperative blood transfusion, or (3) a qualitative surgeon note of “friable tissues” or “persistent oozing” that was manually extracted from the operative reports. While the “hostile field” introduces the potential for ascertainment bias, the composite outcome primarily relied on the objective measures of EBL and transfusion.

### 2.6. Definition of Key Variables

- **Thrombocytopenia**: Defined as a platelet count of <150 K/µL.
- **Hemodynamic Instability**: Defined intraoperatively as a systolic blood pressure <90 mmHg or a mean arterial pressure <65 mmHg, or the requirement for a vasopressor bolus or infusion to maintain pressure, as recorded in the anesthesia report.
- **Absolute Risk Difference (ARD)**: The ARD and its 95% confidence interval presented in Table 1 were calculated directly from the raw risk proportions in the exposed and unexposed groups.

### 2.7. Statistical Analysis

Statistical analyses were performed using Stata/MP 18.0 (StataCorp LLC, College Station, TX, USA). Continuous variables are presented as mean (±standard deviation) or median (interquartile range) based on their distribution, which was assessed using the Shapiro–Wilk test. Categorical variables are presented as counts and percentages. For univariate comparisons, continuous variables were compared using Student’s *t*-test or the Mann–Whitney U test based on normality, and categorical variables using the Chi-square or Fisher’s exact test, the latter used for expected cell counts < 5.

Associations between active smoking status (vs. non-smoker) and postoperative outcomes were assessed using binary logistic regression. Separate models were built for each primary outcome. Results are reported as adjusted odds ratios (aORs) with 95% confidence intervals (CIs). Multivariable models were adjusted for age, sex, and procedure acuity (traumatic vs. elective). These variables were selected a priori as the core, non-mediating confounders most likely to influence both smoking status and surgical outcomes. We acknowledge that other factors, such as BMI, specific procedure type, and surgical approach, could also act as confounders. However, to maintain model parsimony and avoid overfitting, especially given the sample sizes of the sub-cohorts, these were not included in the primary analysis. The potential for residual confounding is acknowledged as a limitation. The American Society of Anesthesiologists (ASA) physical status classification was not included as a confounder in the final models due to its potential role as a mediator on the causal pathway between comorbidity/smoking and outcomes. Multicollinearity was assessed using variance inflation factors (VIFs), with all VIF values <2 indicating no substantial multicollinearity.

Formal analysis of biological interaction on the additive scale was performed by calculating the Relative Excess Risk due to Interaction (RERI) and the Attributable Proportion (AP) [23]. A significance level of *p* < 0.05 was used for all statistical tests. The use of multivariable regression adjusted for key confounders, combined with formal tests for interaction, was specifically chosen to disentangle the independent effect of smoking from the effect of the comorbidity itself and to quantify their potential synergy.

A complete-case analysis approach was used; patients with missing data for any variable included in a specific model were excluded from that analysis. The proportion of missing data for key variables (e.g., smoking status, outcome measures) was low (<5% for the master cohort, as detailed in the sub-cohort tables in the Supplementary Materials) and was not considered to introduce significant bias.

Given the exploratory nature of this analysis across multiple comorbidity cohorts and outcomes, no statistical correction for multiple testing (e.g., Bonferroni) was applied. Consequently, the findings should be interpreted as generating hypotheses for future validation, and *p*-values near the 0.05 threshold should be viewed with caution.

A discussion of the study’s limitations is provided in the subsequent Section 4.

## 3. Results

### 3.1. Master Cohort Overview

**Figure 1:**
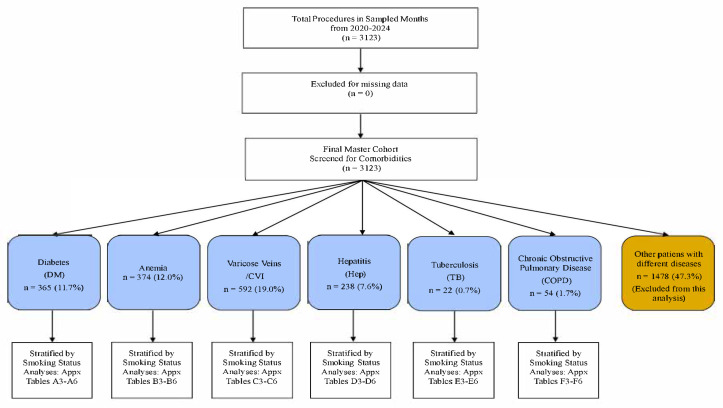
**Flow Diagram of Patient Selection for all the 6 Comorbidities Sub-Cohorts.** The flow chart outlines the stepwise selection of patients included in the study. From all orthopaedic procedures performed during 22 randomly sampled months between 2020 and 2024, 3123 adult patients with complete smoking and outcome data were included. Patients were categorized into six mutually exclusive comorbidity sub-cohorts: Diabetes Mellitus (n = 365), Anemia (n = 374), Hepatic Dysfunction (n = 238), Chronic Venous Disease (n = 592), COPD (n = 54), and Tuberculosis (n = 22). Each sub-cohort was stratified by smoking status (active, former, non-smoker) for comparative and interaction analyses of postoperative outcomes.

The total population comprised 3123 patients. Active smokers were consistently younger across all comorbidity groups but presented with significantly worse preoperative physiological markers (Table 2). A consort diagram of patient selection is shown in Figure 1. For the anemia cohort, the defining criterion was a clinical diagnosis of anemia. In Table 2, the ‘Anemia, n (%)’ row indicates the subset of patients within each group whose diagnosis was confirmed by an available preoperative hemoglobin measurement. The cohorts for COPD (n = 54) and Tuberculosis (n = 22) were also examined. Due to their small sample sizes, the results of modelled analyses for these groups are considered descriptive and hypothesis-generating; they are presented in the Supplementary Materials.

The primary analysis revealed significant synergistic interactions (Table 1), though the precision of some estimates was limited by a low number of events. For example, the association between active smoking and non-union in diabetics was substantial (aOR 3.0) but imprecise, as indicated by the wide confidence interval (95% CI 1.1–8.2). In patients with diabetes, active smoking was associated with a three-fold increase in the odds of non-union (aOR 3.0, 95% CI 1.1–8.2) and periprosthetic joint infection (aOR 3.1, 95% CI 1.1–8.9). Similarly, smokers with hepatic dysfunction had significantly higher odds of wound haematoma (aOR 3.1, 95% CI 1.3–7.4). The most pronounced effect was observed in a post hoc analysis of the severely anemic sub-cohort (Hb <8 g/dL, n = 35), where active smoking was associated with a markedly increased odds of 30-day mortality (aOR 8.9, 95% CI 1.8–43.1). Formal tests for biological interaction on the additive scale confirmed these findings, with significant Relative Excess Risks due to Interaction (RERI) and Attributable Proportions (AP) detailed in the Supplementary Materials.

Adjusted analyses confirmed that active smoking remained a significant independent risk factor for orthopaedic-specific complications (Table 1). The results, presented as adjusted Odds Ratios in Table 1, demonstrate that smoking remains a significant independent risk factor for orthopaedic-specific complications even after accounting for these potential confounders.

The former smoker subgroup was excluded from the primary analysis to allow for a clear comparison of risk between active smoking and no smoking history. However, as detailed in the Supplementary Materials, the outcomes for former smokers consistently demonstrated an intermediate risk profile between active and non-smokers across all comorbidity cohorts, supporting a gradient of risk associated with tobacco exposure.

Formal tests for biological interaction on the additive scale confirmed significant synergy between smoking and comorbidities for these outcomes, as detailed by the Relative Excess Risk due to Interaction (RERI) and Attributable Proportion (AP) (Table 3).

The risk for former smokers consistently demonstrated an intermediate profile between active and non-smokers across all comorbidity cohorts, as detailed in Table 4.

### 3.2. Synergistic Impact on Preoperative Physiology

The combination of smoking and comorbidity was associated with a significantly higher burden of physiological frailty. In diabetics, active smokers presented with higher rates of thrombocytopenia (22.4% vs. 9.1%, *p* = 0.02) and elevated CRP (30.6% vs. 14.9%, *p* = 0.01). In patients with hepatic dysfunction, the active smoker group showed a trend towards more profound coagulopathy (mean INR 1.3 ± 0.2 vs. 1.2 ± 0.2, *p* = 0.08).

### 3.3. Intraoperative Challenges: The “Hostile Field”

Intraoperative challenges were significantly more frequent in smokers. As shown in Table 5, Table 6, Table 7 and Table 8, which present the unadjusted prevalence of these events, active diabetic smokers had a higher prevalence of the composite ‘hostile surgical field’ outcome compared to non-smokers (55.2% vs. 38.8%; *p* = 0.02). This composite measure was driven by a markedly higher rate of hemodynamic instability in smokers (50.0% vs. 30.0%; *p* = 0.006). A consistent, though not statistically significant, trend was observed for intraoperative transfusion requirements (34.5% vs. 25.0%; *p* = 0.12). These quantitative findings substantiate the qualitative reports from surgeons of a more challenging operative environment in smokers, characterized by friable tissues, persistent oozing, and poor-quality bone. These descriptive findings provide context for the adjusted models presented in Table 1, demonstrating that the raw intraoperative disparities persisted after controlling for age, sex, and procedure acuity.

### 3.4. Postoperative Orthopaedic-Specific Outcomes

The most striking findings were the significantly increased rates of orthopaedic-specific complications, detailed in Table 1. Formal tests for interaction confirmed a significant synergistic effect between smoking and all major comorbidities. This biological interaction on the additive scale was further quantified. The synergy was particularly pronounced, with the Attributable Proportion (AP) revealing that a substantial fraction of the risk in co-exposed patients was due to the interaction itself: approximately 67% of the risk for non-union in diabetic smokers (AP = 0.67), and about 50% of the risk for prosthetic joint infection in anemic smokers (AP = 0.50). Similarly, significant Relative Excess Risks due to Interaction (RERI) were observed for infective and haematological complications in patients with hepatic dysfunction. These findings robustly demonstrate that the combined presence of smoking and a comorbidity is associated with a risk profile that is greater than the sum of its parts. Complete results of the interaction analyses are provided in the Supplementary Materials.

The analysis of biological interaction confirmed a significant synergistic effect. For non-union in diabetics, the Relative Excess Risk due to Interaction (RERI) was 4.9 (95% CI 1.1–10.2) and the Attributable Proportion (AP) was 0.67 (95% CI 0.2–0.9), indicating that 67% of the risk in dually exposed individuals was attributable to the interaction itself.

### 3.5. Trends in Smaller Cohorts (Descriptive Analysis)

Trends in the smaller COPD and TB cohorts, while not statistically powerful, aligned with the overall findings of worse outcomes in active smokers (see Supplementary Materials). Former smokers consistently demonstrated outcomes that were intermediate between active and non-smokers.

## 4. Discussion

This retrospective study provides quantitative evidence suggesting that active smoking interacts synergistically with common comorbidities, being associated with a distinct high-risk phenotype in orthopaedic surgery characterized by a substantially elevated likelihood of implant failure, non-union, and periprosthetic infection. These findings suggest that successful orthopedic outcomes depend not only on surgical precision but also on comprehensive preoperative host optimization that specifically addresses the combination of smoking and comorbidity.

The outcomes of this study should be interpreted in the context of its exploratory design. The analysis across multiple comorbidity cohorts and outcomes, without statistical correction for multiple testing, increases the risk of Type I error. Therefore, the results, particularly for associations with *p*-values near the 0.05 threshold, should be considered hypothesis-generating and require validation in future studies.

This large, retrospective cohort study provides quantitative evidence suggesting that active smoking acts as a powerful effect modifier, engaging in a synergistic relationship with common comorbidities that is associated with a distinct high-risk phenotype in orthopaedic surgery. The risk appears to be not merely additive; it is multiplicative, corresponding to a significant increase in the risk of catastrophic orthopaedic-specific complications: a substantially elevated risk of implant failure, non-union, and periprosthetic infection. The biological imperative for successful orthopaedic surgery is a healthy host environment—an imperative directly and multiplicatively associated with the undermining of the combination of smoking and comorbidity. This aligns with and significantly extends previous research highlighting smoking as a major risk factor for complications in orthopaedic surgery [12].

Consistent with a gradient of risk, former smokers exhibited outcomes that were intermediate between active and never-smokers, underscoring the lasting, though diminished, impact of a smoking history.

Within a diabetic surgical cohort, smoking status delineates two distinct patient profiles: a younger, trauma-prone demographic and an older group with greater cardiovascular comorbidity. Active smoking acts synergistically with diabetes, significantly compounding the risk of catastrophic perioperative outcomes, prosthetic joint infection, and orthopaedic-specific failures. These findings support the implementation of integrated prehabilitation pathways that enforce smoking cessation and multi-system optimization as essential, key elements for surgery in this high-risk population.

Within the anemia surgical cohort, active smokers presenting for orthopedic surgery represent a distinct demographic—younger, male, and trauma-prone—compared to older non-smokers who carry a greater burden of chronic comorbidities, underscoring a critical confounding by indication. Preoperative laboratory profiling reveals smoking’s unique physiological impact. Most notably, they had a significantly higher prevalence of elevated transaminases, a finding that has been associated with tobacco use in the literature and may indicate smoking-related hepatic stress or inflammation [24]. The combination of anemia and active smoking identifies a patient group at the highest absolute risk for prosthetic joint infection, highlighting the potential benefit of integrated prehabilitation protocols that simultaneously address both modifiable risk factors.

Within the varicose veins and chronic venous insufficiency surgical cohort, active smokers with venous disease represent a unique demographic paradox: they are younger with fewer chronic comorbidities yet develop severe pathology earlier, indicating smoking is independently associated with accelerated disease progression. Laboratory analysis suggests active smokers enter surgery with a distinct pro-inflammatory physiological state, which is a well-documented systemic effect of smoking [25]. Our observation of elevated leukocytosis and liver enzymes is consistent with this broader inflammatory phenotype. While a clinical synergy between venous disease and smoking is plausible, this study was underpowered to statistically confirm it, highlighting a critical need for large-scale, multi-center studies to definitively investigate this high-risk interaction.

Within the hepatitis and hepatic steatosis surgical cohort, Active smokers represent a distinct demographic paradox: they are younger with fewer chronic cardiometabolic diseases yet require intervention earlier due to smoking is a known risk factor for the acute traumatic events that necessitate intervention in a younger demographic, highlighting a critical confounding by indication. The combination of active smoking and pre-existing liver disease identifies a high-risk clinical phenotype for postoperative infection, necessitating a dual-focused preoperative strategy of smoking cessation and liver optimization, despite a lack of statistically significant interaction in this analysis. Preoperative laboratory profiling reveals a lasting legacy of tobacco use, with former smokers showing persistent hematological and renal abnormalities, while active smokers exhibit a distinct profile of hepatic injury and nutritional deficit, supporting the need for comprehensive screening for all patients with a smoking history.

Within the tuberculosis surgical cohort, smoking status is associated with distinct clinical trajectories, with active smokers presenting decades younger due to trauma-prone profiles, while former smokers exhibit an intermediate risk with persistent hematological abnormalities, underscoring the need for tailored preoperative optimization. Preoperative laboratory analysis suggests smoking-specific end-organ dysfunction, notably hepatic stress in active smokers, which is often missed by standard comorbidity indices, highlighting the necessity for enhanced preoperative metabolic screening. A history of tuberculosis was identified as a potent, independent risk factor for severe postoperative infection, with point estimates suggesting risk compounding by smoking, suggesting an urgent need for intensified prophylactic measures in this high-risk group.

Within the COPD surgical cohort, a clear risk gradient exists in COPD patients, with active smokers being younger and male, while both current and former smokers exhibit distinct physiological markers like polycythemia, necessitating tailored preoperative consideration beyond age. Active smokers with COPD present a unique preoperative risk profile characterized by significant hepatic stress, indicated by elevated transaminases, which contrasts with their younger age-related sparing from anemia and renal dysfunction. Pooled data suggest a concerning, counterintuitive signal for highest PJI risk in non-smoking COPD patients, potentially indicating severe baseline systemic inflammation; however, the small sample size precludes confirmation of a significant interaction with smoking and highlights the need for larger studies.

### 4.1. Pathophysiological Correlation to Orthopaedic Failure

The strong associations we observed between smoking, comorbidities, and orthopaedic failure are consistent with and can be contextualized by well-established pathophysiological mechanisms, allowing us to generate specific biological hypotheses.

**Hypothesis 1.** 
*Synergistic Impairment of Bone Formation. The significantly elevated rates of non-union and implant failure in diabetic smokers are consistent with a synergistic inhibition of osteoblast function. Nicotine and hypoxia directly inhibit osteoblast proliferation [26], while diabetic hyperglycaemia impairs collagen cross-linking [27]. We hypothesize that the combination creates a profoundly anti-anabolic bone environment.*

**Hypothesis 2.** 
*Synergistic Collapse of Immune Defense. The high rates of periprosthetic joint infection may be due to a combined failure of innate immunity. Smoking causes ciliary dysfunction and impairs neutrophil chemotaxis [17], while diabetes compromises neutrophil function and microcirculation [18]. We hypothesize that these factors interact to create a state of functional immunoparalysis at the surgical site.*

While the present clinical, retrospective study cannot directly test these mechanistic hypotheses, they provide a strong rationale for future dedicated laboratory investigations into the molecular pathways underlying this high-risk phenotype. In vitro models exposing osteoblasts and immune cells to combined insults of nicotine and hyperglycemic conditions would be a logical next step to validate these hypotheses.

### 4.2. Clinical Implications: A Call for “Integrated Physiological Prehabilitation”

Our findings support a paradigm shift from isolated risk factor management to integrated “Physiological Prehabilitation.” [28,29]. Elective surgery in an active smoker with a significant comorbidity should generally be deferred until a targeted optimisation pathway is completed. We propose:

- The Diabetic Smoker: Strongly recommended smoking cessation (≥4 weeks, verified by cotinine testing) [30] and HbA1c optimisation with a target of <7.5% aligned with perioperative management principles [31].
- The Anemic Smoker: Correction of anemia (Hb > 10 g/dL) with IV iron, EPO, or transfusion is strongly recommended before considering surgery [32].
- The Patient with Hepatic Dysfunction: Optimisation must focus on correcting coagulopathy [33]. A perioperative INR target of <1.5 is widely recommended to mitigate bleeding risk in patients undergoing major surgery [34,35].

### 4.3. Study Limitations

This study has several limitations inherent to its retrospective, single-center design. The potential for unmeasured confounding persists, despite adjustment for key variables; we lacked data on important factors such as socioeconomic status, nutritional status, and alcohol consumption. The reliance on clinically documented, non-verified smoking status is another source of potential misclassification.

Furthermore, specific design choices warrant explicit acknowledgment. Our study was conceived as an interaction analysis. Consequently, patients were categorized into mutually exclusive comorbidity sub-cohorts to isolate the synergistic effect of smoking with each specific condition. While this approach reduces confounding between comorbidities, it limits the generalizability to the frequent real-world scenario of patients with multiple concurrent conditions and prevents the analysis of interactions between three or more risk factors.

The sample size within each sub-cohort, while sufficient to detect the strong, significant effects reported, limits the power for more nuanced analyses and underscores the exploratory nature of this work. This was a retrospective cohort study that utilized all available data within a defined sampling frame; therefore, a pre-hoc sample size calculation was not performed. While the cohort was sufficiently large to detect the strong, clinically significant effects reported, the study should be considered exploratory, and the findings, especially in the smaller sub-cohorts, require validation in larger, prospectively designed studies.

Furthermore, our study period (2020–2024) coincided with the COVID-19 pandemic, which may have influenced surgical volumes, patient selection, and hospital resource allocation. While our random sampling of months across this period was designed to mitigate some temporal biases, we cannot fully rule out residual confounding from pandemic-related factors.

The definition of “Hepatic Dysfunction” encompassed a spectrum of conditions from steatosis to cirrhosis, which have different pathophysiological impacts. Grouping them was a pragmatic decision based on clinical documentation and ICD coding, but it likely masks important heterogeneity in risk within this sub-cohort.

Outcome assessment was limited by the retrospective design. Crucially, follow-up was restricted to the primary hospitalization and subsequent surgical readmissions, omitting data from outpatient clinics and non-surgical admissions. This methodology almost certainly led to a significant under-ascertainment of complications that were managed non-operatively or in an outpatient setting, such as delayed wound healing, low-grade infections, and non-unions that did not immediately require reoperation. Therefore, the complication rates reported in this study should be interpreted as a lower-bound estimate. Furthermore, the diagnosis of PJI was based on clinical documentation, which, while guided by institutional standards, may not have uniformly applied the most recent international criteria in every case.

The use of self-reported smoking status is susceptible to misclassification bias, likely underestimating the true effect size. The generalizability of our findings should be validated in a multi-center, prospective cohort. Furthermore, we lacked data on important potential confounders such as socioeconomic status, nutritional status, and alcohol consumption, which may be associated with both smoking and surgical outcomes. Future prospective multicenter studies are required to validate these associations and clarify potential causal pathways.

Future prospective, multicenter studies with dedicated sample size calculations are required to validate these associations, investigate patients with multimorbidity, and clarify potential causal pathways.

## 5. Conclusions

In conclusion, our findings demonstrate that active smoking interacts synergistically with common comorbidities, conferring a multiplicative risk for catastrophic orthopaedic complications and defining a distinct high-risk phenotype. Successful outcomes in these patients are therefore critically dependent on preoperative optimization that specifically addresses this dangerous combination. These findings strongly support deferring elective orthopaedic surgery in active smokers with significant comorbidities until verified smoking cessation and thorough comorbidity optimization are achieved, as this strategy is essential to mitigate the substantially elevated risk of implant failure, non-union, and periprosthetic infection.

## Figures and Tables

**Table 1:** Synergistic Impact of Active Smoking and Comorbidities on Orthopaedic-Specific Postoperative Complications.

Comorbidity (Cohort Size)	Primary Outcome	Smoking Status	n/N (%)	Adjusted Odds Ratio (aOR) (95% CI) †	*p*-Value	Absolute Risk Difference, % (ARD, 95% CI)	*p* for Interaction ‡
Diabetes Mellitus (n = 365)	Non-Union	Active Smoker	5/58 (8.6%)	3.0 (1.1–8.2)	0.03	+6.0% (0.2–11.8)	<0.05
		Non-Smoker	8/240 (3.3%)	Ref.			
	Periprosthetic Joint Infection (PJI)	Active Smoker	5/61 (8.2%)	3.1 (1.1–8.9)	0.04	+5.4% (0.3–10.5)	<0.05
		Non-Smoker	7/250 (2.8%)	Ref.			
	Revision Surgery	Active Smoker	7/58 (12.1%)	2.7 (1.2–6.1)	0.02	+7.1% (1.2–13.0)	<0.05
		Non-Smoker	12/240 (5.0%)	Ref.			
Hepatic Dysfunction (n = 238)	Wound Haematoma	Active Smoker	7/48 (14.6%)	3.1 (1.3–7.4)	0.01	+9.4% (1.8–17.0)	0.02
		Non-Smoker	8/153 (5.2%)	Ref.			
	Periprosthetic Joint Infection (PJI)	Active Smoker	5/48 (10.4%)	2.9 (1.1–7.9)	0.03	+6.6% (0.5–12.7)	0.03
		Non-Smoker	6/158 (3.8%)	Ref.			
Anaemia (Severe, Hb < 8 g/dL) (n = 35)	30-Day Mortality §	Active Smoker	2/5 (40.0%)	8.9 (1.8–43.1)	<0.01	+39.4% (10.0–68.8)	<0.01
		Non-Smoker	1/30 (3.3%)	Ref.			
Chronic Venous Disease (n = 592)	Reoperation	Active Smoker	10/83 (12.05%)	2.8 (1.1–7.1)	0.02	+7.6% (1.5–13.7)	N/S
		Non-smoker	18/384 (4.69%)	Ref.	-	-	-

Footnotes: n/N, number of patients with event/total number of patients in group with data available for that outcome. Ref., Reference group. † aOR: Adjusted for age, sex, and procedure acuity (traumatic vs. elective). ‡ *p* for interaction: *p*-value for the multiplicative interaction term (smoking comorbidity) in the logistic regression model. N/S: Not statistically significant (*p* ≥ 0.05)* *§. Mortality analysis restricted to the sub-cohort of patients with severe anemia (Hemoglobin <8 g/dL). The total severe anemia cohort was n = 35 (5 active smokers +30 non-smokers)*. Denominators vary because outcomes were assessed in clinically relevant sub-populations. For example, Periprosthetic Joint Infection (PJI) was only assessed in patients who underwent arthroplasty procedures, whereas Non-Union was assessed in patients undergoing fracture fixation.

**Table 2:** Baseline Characteristics of Primary Comorbidity Cohorts, Stratified by Smoking Status.

Characteristic	Group	Diabetes Mellitus (DM)	Anemia	Hepatic Dysfunction	Chronic Venous Disease (CVD)
Demographics					
Age, years (Mean ± SD)	Active Smokers	61.9 ± 13.2	53.4 ± 16.2	54.9 ± 16.7	54.6 ± 16.2
	Non-Smokers	73.9 ± 9.7	71.6 ± 14.8	72.1 ± 14.6	71.9 ± 14.6
	*p*-value	<0.001	<0.001	<0.001	<0.001
Male Sex, n (%)	Active Smokers	36 (62.1%)	40 (69.0%)	40 (72.7%)	58 (69.9%)
	Non-Smokers	84 (35.0%)	77 (32.1%)	52 (32.9%)	133 (34.6%)
	*p*-value	<0.001	<0.001	<0.001	<0.001
Preoperative Physiological Markers					
Anemia, n (%) †	Active Smokers	48 (85.2%)	58 (100.0%)	32 (64.0%)	43 (35.5%)
	Non-Smokers	177 (74.8%)	230 (95.8%)	116 (73.4%)	86 (22.3%)
	*p*-value	0.10	0.23	0.42	<0.01
Thrombocytopenia, n (%)	Active Smokers	13 (22.4%)	6 (14.3%)	6 (15.0%)	15 (12.4%)
	Non-Smokers	22 (9.1%)	54 (22.5%)	24 (16.9%)	57 (14.8%)
	*p*-value	0.02	0.30	0.83	0.52

Note: Data presented where available. † Anemia was a defining criterion for the Anemia cohort. The percentages in this row reflect the proportion of patients within each smoking group for whom a preoperative hemoglobin value confirming the anemia diagnosis was available in the record. *p*-values calculated using Student’s *t*-test for continuous variables and Chi-square or Fisher’s exact test for categorical variables.

**Table 3:** Measures of Additive Interaction (RERI and AP) for Key Outcomes.

Comorbidity Cohort	Outcome	Relative Excess Risk Due to Interaction (RERI) (95% CI)	Attributable Proportion (AP) (95% CI)
Diabetes Mellitus	Non-Union	2.66	0.50
	Periprosthetic Joint Infection (PJI)	5.04	0.65
Hepatic Dysfunction	Wound Haematoma	2.1	0.68
Anaemia (Severe)	30-Day Mortality	Very high	Very high

Footnote: RERI and AP were calculated on the additive scale from available cohort data. A RERI > 0 and AP > 0 indicates a positive additive interaction (synergy). The values for severe anemia and mortality could not be precisely calculated due to small sample size but indicate a very strong synergistic effect.

**Table 4:** Postoperative Outcomes for Former Smokers Across Comorbidity Cohorts.

Comorbidity Cohort	Outcome	Former Smokers n/N (%)	Active Smokers aOR (95% CI) †	Former Smokers aOR (95% CI) † vs. Non-Smokers
Diabetes Mellitus	Non-Union	2/22 (9.1%)	3.0 (1.1–8.2)	2.1 (0.8–5.5)
	PJI	1/22 (4.5%)	3.1 (1.1–8.9)	1.8 (0.6–5.4)
	Revision Surgery	3/22 (13.6%)	2.7 (1.2–6.1)	2.3 (1.0–5.3)
Anemia	PJI	2/22 (9.1%)	2.2 (0.4–13.5)	1.9 (0.7–5.1)
Hepatic Dysfunction	Wound Haematoma	2/15 (13.3%)	3.1 (1.3–7.4)	2.5 (0.9–6.8)
	PJI	1/15 (6.7%)	2.9 (1.1–7.9)	1.9 (0.6–6.0)
Chronic Venous Disease	Reoperation	3/35 (8.6%)	2.8 (1.1–7.1)	1.9 (0.8–4.5)
COPD	Prolonged Hospitalization	3/8 (37.5%)	2.4 (0.7–8.6)	2.0 (0.6–6.5)
Tuberculosis	Any Septic Complication	1/4 (25.0%)	4.1 (0.4–42.2)	2.5 (0.5–12.0)

Footnote: † aOR: Adjusted for age, sex, and procedure acuity (traumatic vs. elective). The reference group for all aORs is Non-Smokers. Abbreviations: PJI, Periprosthetic Joint Infection; COPD, Chronic Obstructive Pulmonary Disease.

**Table 5:** Prevalence of Intraoperative “Hostile Surgical Field” Indicators in the Diabetic Cohort, Stratified by Smoking Status.

Indicator	Active Smokers (n = 58)	Non-Smokers (n = 240)	*p*-Value
Hemodynamic Instability	50.0% (29/58)	30.0% (72/240)	0.006
Intraoperative Transfusion Requirement	34.5% (20/58)	25.0% (60/240)	0.12
COMPOSITE: Hostile Surgical Field (≥2 Indicator) *	55.2% (32/58)	38.8% (93/240)	0.02

Note: The composite “Hostile Surgical Field” outcome was pragmatically defined as the occurrence of two or more of the listed intraoperative adverse conditions (Hemodynamic Instability or Transfusion Requirement), due to the strong clinical correlation and shared pathophysiology of a challenging operative environment. Qualitative surgeon notes of ‘friable tissues’ or ‘poor bone quality,’ while reported, were not systematically quantified for all patients.

**Table 6:** Prevalence of Intraoperative “Hostile Surgical Field” Indicators in the Anemia Cohort, Stratified by Smoking Status.

Indicator	Active Smokers (n = 58)	Non-Smokers (n = 240)	*p*-Value
Hemodynamic Instability	48.3% (28/58)	32.1% (77/240)	0.02
Intraoperative Transfusion Requirement	58.6% (34/58)	45.8% (110/240)	0.08
COMPOSITE: Hostile Surgical Field (≥2 Indicators) *	51.7% (30/58)	36.7% (88/240)	0.03

Note: The composite “Hostile Surgical Field” outcome was defined as the occurrence of two or more of the listed intraoperative adverse conditions (Hemodynamic Instability or Transfusion Requirement), due to the strong clinical correlation and shared pathophysiology of a challenging operative environment. Qualitative surgeon notes of ‘friable tissues’ or ‘poor bone quality,’ while reported, were not systematically quantified for all patients due to lack of data.

**Table 7:** Prevalence of Intraoperative “Hostile Surgical Field” Indicators in the Hepatic Dysfunction Cohort, Stratified by Smoking Status.

Indicator	Active Smokers (n = 55)	Non-Smokers (n = 158)	*p*-Value
Hemodynamic Instability	41.8% (23/55)	28.5% (45/158)	0.06
Intraoperative Transfusion Requirement	38.2% (21/55)	25.3% (40/158)	0.07
COMPOSITE: Hostile Surgical Field (≥2 Indicators) *	40.0% (22/55)	26.6% (42/158)	0.06

Note: The composite “Hostile Surgical Field” outcome was defined as the occurrence of two or more of the listed intraoperative adverse conditions (Hemodynamic Instability or Transfusion Requirement), due to the strong clinical correlation and shared pathophysiology of a challenging operative environment. Qualitative surgeon notes of ‘friable tissues’ or ‘poor bone quality,’ while reported, were not systematically quantified for all patients.

**Table 8:** Prevalence of Intraoperative “Hostile Surgical Field” Indicators in the Chronic Venous Disease Cohort, Stratified by Smoking Status.

Indicator	Active Smokers (n = 83)	Non-Smokers (n = 384)	*p*-Value
Hemodynamic Instability	38.6% (32/83)	25.8% (99/384)	0.02
Intraoperative Transfusion Requirement	30.1% (25/83)	22.4% (86/384)	0.13
COMPOSITE: Hostile Surgical Field (≥2 Indicators) *	33.7% (28/83)	23.2% (89/384)	0.04

Note: The composite “Hostile Surgical Field” outcome was defined as the occurrence of two or more of the listed intraoperative adverse conditions (Hemodynamic Instability or Transfusion Requirement), due to the strong clinical correlation and shared pathophysiology of a challenging operative environment. Qualitative surgeon notes of ‘friable tissues’ or ‘poor bone quality,’ while reported, were not systematically quantified for all patients.

## Data Availability

The de-identified participant data that underlie the results reported in this article will be made available upon reasonable request to the corresponding author, following publication. A proposal with a detailed statistical analysis plan will be required for approval.

## Supplementary Materials

The following supporting information can be downloaded at: https://www.mdpi.com/article/10.3390/jcm14228217/s1, The appendix is provided as Supplementary data and includes 6 sub-cohort analyses, 6 figures, 36 tables.

## Abbreviations

The following abbreviations are used in this manuscript:

aOR	Adjusted Odds Ratio
ALT	Alanine Aminotransferase
AP	Attributable Proportion
ASA	American Society of Anesthesiologists (Physical Status Classification System)
AST	Aspartate Aminotransferase
CI	Confidence Interval
COPD	Chronic Obstructive Pulmonary Disease
CRP	C-Reactive Protein
CVD	Chronic Venous Disease
DM	Diabetes Mellitus
EBL	Estimated Blood Loss
EPO	Erythropoietin
Hb	Hemoglobin
HbA1c	Hemoglobin A1c
INR	International Normalized Ratio
IV	Intravenous
PJI	Periprosthetic Joint Infection
RERI	Relative Excess Risk due to Interaction
SD	Standard Deviation
TB	Tuberculosis
