# Supplementary material for: Smoking Amplifies Comorbidity-Associated Risk in Orthopaedic Surgery: A Multiplicative Interaction
Source: J Clin Med. 2025 Nov 19;14(22):8217. doi: 10.3390/jcm14228217 (PMC12653757; doi:10.3390/jcm14228217)
Supplement: Supplementary file 1 [file jcm-14-08217-s001.zip › jcm-3980122-supplementary.pdf]

TITLE: Smoking Amplifies Comorbidity-Associated Risk in Orthopaedic Surgery: A Multiplicative Interaction

AUTHORS:

Edith Simona Ianoși*[1], Daria-Maria Roșu*[2], Arpad Solyom[3], Bianca Liana Grigorescu[4], Mara Vultur[1], Maria Beatrice Ianoși[5]

***These authors contributed equally to this paper**

AFFILIATIONS:

[1] Department of Pulmonology, University of Medicine, Pharmacy, Technology and Sciences "George Emil Palade" of Târgu Mureș, 540139 Târgu Mures, Romania, edith.ianosi@umfst.ro(E.S.-I.),mara.vultur@umfst.ro (VM)

[2] University of Medicine, Pharmacy, Science and Technology "George Emil Palade" of Târgu Mureș, 540139 Târgu Mures, Romania, rosu.daria-maria.24@stud.umfst.ro; (D.M.-R.)

[3]Department of Orthopaedic and Traumatology Surgery, University of Medicine, Pharmacy, Science and Technology "George Emil Palade" of Târgu Mureș, 540139 Târgu Mures 540139 Târgu Mures, Romania, arpad.solyom@umfst.ro; (A.S.)

[4]Department of Anaesthesiology and Intensive Care, University of Medicine, Pharmacy, Science and Technology "George Emil Palade" of Târgu Mureș, 540139 Târgu Mures, Romania, bianca.grigorescu@umfst.ro; (B.L.-G.)

[5]Pulmonology Clinic, Mures, County Clinical Hospital, Târgu Mures, 540011 Târgu Mures, Romania; ianosi.maria-beatrice@stud18.umfst.ro; (M.B.-I.)

**Correspondence*: email: rosu.daria-maria.24@stud.umfst.ro

WORD COUNT: 17274

FIGURE COUNT: 6

TABLE COUNT: 36

SUPPLEMENTARY APPENDIX: The appendix is provided as Supplementary data and includes 6 sub-cohort analyses, 6 figures, 36 tables.

KEYWORDS: Tobacco Use; Postoperative Complications; Orthopaedic Procedures; Diabetes Mellitus; Anemia; Venous Insufficiency; Hepatitis; Tuberculosis; COPD; Risk Assessment

AUTHOR CONTRIBUTIONS: Conceptualization, E.S.-I., D.M.-R., A.S., B.L.-G., V.M., M.B.-I.; Methodology, E.S.-I, D.M.-R., A.S., B.L.-G, V.M, M.B.-I ; Software, E.S.-I, D.M.-R., A.S., B.L.-G, V.M., M.B.-I; Validation, E.S.-I, D.M.-R., A.S., B.L.-G., V.M, M.B.-I.; Formal Analysis E.S.-I., D.M.-R., A.S., B.L.-G., V.M., M.B.-I; Investigation, E.S.-I., D.M.-R., A.S., B.L.-G., V.M., M.B.-I.; Resources E.S.-I., D.M.-R., B.L.-G., M.B.-I.; Data Curation, E.S.-I., D.M.-R., A.S., B.L.-G., V.M., M.B.-I.; Writing—Original Draft Preparation, E.S.-I., D.M.-R., B.L.-G., M.B.-I.; Writing—Review and Editing, E.S.-I., D.M.-R., M.B.-I.; Visualization, E.S.-I., D.M.-R.; Supervision, E.S.-I., D.M.-R; Project Administration, E.S.-I., D.M.-R.

CONFLICTS OF INTEREST: The authors declare no conflicts of interest.

FUNDING: This research received no external funding.

DATA SHARING: The de-identified participant data that underlie the results reported in this article will be made available upon reasonable request to the corresponding author, following publication. A proposal with a detailed statistical analysis plan will be required for approval.

INSTITUTIONAL REVIEW BOARD STATEMENT: The study was conducted in accordance with the Decla-ration of Helsinki, and approved by the Ethics Committee of the County Emergency Clinical Hospital of Tîrgu Mureș (Protocol number Ad. 14346 and date of the approval 28.05.2025).

INFORMED CONSENT STATEMENT: The requirement for informed consent was waived due to the retrospective, observational nature of the study.

ACKNOWLEDGMENTS: The authors would like to extend their sincere gratitude to the administrative and data management staff of the County Emergency Clinical Hospital of Tîrgu Mureș for their invaluable assistance in facilitating the data retrieval process for this study.

We would also like to thank the medical and research community at the University of Medicine, Pharmacy, Science and Technology "George Emil Palade" for their ongoing support and scholarly discourse.

During the preparation of this manuscript, the authors used OpenAI for the purposes of initial grammar and clarity checks on early drafts. The authors have thoroughly reviewed, edited, and take full responsibility for the entire content of this publication.

Abstract

TITLE

Smoking Amplifies Comorbidity-Associated Risk in Orthopaedic Surgery: A Multiplicative Interaction

BACKGROUND

The success of orthopaedic surgery is fundamentally biological, yet the synergistic effect of smoking and comorbidities on surgical outcomes is not well quantified. We **hypothesised** that active smoking multiplies the risk conferred by common comorbidities.

METHODS

In this retrospective cohort study, we analysed 3,123 orthopaedic procedures from 2020 to 2024. Patients were stratified by comorbidity (diabetes, anemia, hepatic dysfunction, chronic venous disease) and smoking status. Primary outcomes were orthopaedic-specific complications, including non-union, periprosthetic joint infection (PJI), and revision surgery. We used multivariate logistic regression to calculate adjusted odds ratios (aORs) and formal tests for interaction to quantify synergy.

RESULTS

A significant synergistic effect was observed. In analyses adjusted for age, sex, and procedure acuity, diabetic smokers had significantly higher rates of non-union (8.6% vs. 3.3%; aOR 3.0, 95% CI 1.1–8.2), periprosthetic joint infection (8.2% vs. 2.8%; aOR 3.1, 95% CI 1.1–8.9), and revision surgery (12.2% vs. 5.0%; aOR 2.7, 95% CI 1.2–6.1). Significant interaction effects confirmed this synergy. Smokers with hepatic dysfunction had higher haematoma rates, while smoking with severe anemia was associated with markedly increased mortality (5.0%; aOR 8.9). Former smokers' outcomes were consistently intermediate between active and non-smokers. For example, in the diabetic cohort, the adjusted odds of non-union were elevated for both former (aOR 2.1, 95% CI 0.8–5.5) and active smokers (aOR 3.0, 95% CI 1.1–8.2) compared to non-smokers (reference), demonstrating a gradient of risk. A complete presentation of outcomes for former smokers across all cohorts is provided in Table 8.

CONCLUSIONS

Smoking is associated with a multiplicative increase in comorbidity risk, creating a distinct high-risk phenotype that severely compromises healing. These findings strongly support that verified smoking cessation should be a foundational component of preoperative optimisation before elective orthopaedic surgery.

Table of Contents

TITLE: 1

AUTHORS: Edith Simona Ianoși[*1], Daria-Maria Roșu[*2], Arpad Solyom[3], Bianca Liana Grigorescu[4], Mara Vultur[1], Maria Beatrice Ianoși[5] 1

WORD COUNT: 17283 2

FIGURE COUNT: 6 2

TABLE COUNT: 36 2

SUPPLEMENTARY APPENDIX: The appendix is provided as Supplementary data and includes 6 sub-cohort analyses, 6 figures, 36 tables. 2

KEYWORDS: Tobacco Use; Postoperative Complications; Orthopaedic Procedures; Diabetes Mellitus; Anemia; Venous Insufficiency; Hepatitis; Tuberculosis; COPD; Risk Assessment 2

AUTHOR CONTRIBUTIONS: Conceptualization, E.S.-I., D.M.-R., A.S., B.L.-G., V.M., M.B.-I.; Methodology, E.S.-I, D.M.-R., A.S., B.L.-G, V.M, M.B.-I ; Software, E.S.-I, D.M.-R., A.S., B.L.-G, V.M., M.B.-I; Validation, E.S.-I, D.M.-R., A.S., B.L.-G., V.M, M.B.-I.; Formal Analysis E.S.-I., D.M.-R., A.S., B.L.-G., V.M., M.B.-I; Investigation, E.S.-I., D.M.-R., A.S., B.L.-G., V.M., M.B.-I.; Resources E.S.-I., D.M.-R., B.L.-G., M.B.-I.; Data Curation, E.S.-I., D.M.-R., A.S., B.L.-G., V.M., M.B.-I.; Writing—Original Draft Preparation, E.S.-I., D.M.-R., B.L.-G., M.B.-I.; Writing—Review and Editing, E.S.-I., D.M.-R., M.B.-I.; Visualization, E.S.-I., D.M.-R.; Supervision, E.S.-I., D.M.-R; Project Administration, E.S.-I., D.M.-R. 2

CONFLICTS OF INTEREST: The authors declare no conflicts of interest. 2

FUNDING: This research received no external funding. 2

DATA SHARING: The de-identified participant data that underlie the results reported in this article will be made available upon reasonable request to the corresponding author, following publication. A proposal with a detailed statistical analysis plan will be required for approval. 2

INSTITUTIONAL REVIEW BOARD STATEMENT: The study was conducted in accordance with the Decla-ration of Helsinki, and approved by the Ethics Committee of the County Emergency Clinical Hos-pital of Tîrgu Mureș (Protocol number Ad. 14346 and date of the approval 28.05.2025). 3

INFORMED CONSENT STATEMENT: The requirement for informed consent was waived due to the retrospective, observational nature of the study. 3

ACKNOWLEDGMENTS: The authors would like to extend their sincere gratitude to the administrative and data management staff of the County Emergency Clinical Hospital of Tîrgu Mureș for their invaluable assistance in facilitating the data retrieval process for this study. 3

We would also like to thank the medical and research community at the University of Medicine, Pharmacy, Science and Technology "George Emil Palade" for their ongoing support and scholarly discourse. 3

During the preparation of this manuscript, the authors used OpenAI for the purposes of initial grammar and clarity checks on early drafts. The authors have thoroughly reviewed, edited, and take full responsibility for the entire content of this publication. ... 3
Abstract ... 4
TITLE ... 4
Appendix: Overall Statistical Methods ... 9
Supplementary Appendix Text S1: Detailed Sampling Methodology and Cohort Construction ... 11
Appendix A: The Diabetes Mellitus (DM) Sub-Cohort (n=365) ... 12
Contents ... 12
Key to Abbreviations: ... 13
Narrative Summaries for Diabetes Mellitus Sub-Cohort ... 14
Appendix Figure A1: Flow Diagram of Patient Selection for the Diabetes Mellitus Sub-Cohort ... 15
Appendix Table A1: Annual Distribution of the Overall Cohort and Diabetes Mellitus Sub-Cohort ... 17
Appendix Table A2: Smoking Status Distribution within the DM Cohort (n=365) ... 18
Appendix Table A3: Baseline Characteristics of the Study Cohort with Diabetes Mellitus, by Smoking Status ... 19
Appendix Table A4: Preoperative Laboratory Profile Stratified by Smoking Status ... 21
Appendix Table A5: Adjusted Associations Between Active Smoking and Postoperative Outcomes ... 22
Appendix Table A6: Risk of Prosthetic Joint Infection (PJI) by Diabetes and Smoking Status ... 24
Appendix Table A7: Illustrative Patient Case Summaries from the DM Cohort (2020-2024) ... 26
Appendix Text A1: Sampling Methodology Note ... 30
Appendix Text A2: Sampling Statistical Methods ... 30
Appendix B: The Anemia Sub-Cohort (n=374) ... 31
Contents ... 31
Key to Abbreviations: ... 32
Narrative Summaries for Anemia Sub-Cohort ... 33

Appendix Figure B1: Flow Diagram of Patient Selection for the Anemia Sub-Cohort 34
Appendix Table B1: Annual Distribution of the Overall Cohort and Anemia Sub-Cohort 36
Appendix Table B2: Smoking Status Distribution within the Anemia Cohort (n=374) 37
Appendix Table B3: Baseline Characteristics and Pre-operative Laboratory Findings of the Anemia Cohort by Smoking Status 38
Appendix Table B4: Prevalence of Preoperative Laboratory Abnormalities by Smoking Status 41
Appendix Table B5: Framework for Unadjusted Associations Between Active Smoking and Postoperative Outcomes 43
Appendix Table B6: Combined Cohort Analysis: RERI and AP for the Interaction Between Anemia and Smoking Status on PJI Risk 44
Appendix Table B7: Illustrative Patient Case Summaries from the Anemia Cohort (2020-2024) 46
Appendix Text B1: Sampling Methodology Note 50
Appendix Text B2: Sampling Statistical Methods 50
Appendix C: The Varicose Veins (VV) & Chronic Venous Insufficiency (CVI) Sub-Cohort (n=592) 51
Contents 51
Key to Abbreviations: 52
Narrative Summaries for Varicose Veins and Chronic Venous Insufficiency Sub-Cohort 54
Appendix Figure C1: Flow Diagram of Patient Selection for the Varicose Veins & Chronic Venous Insufficiency Sub-Cohort 55
Appendix Table C1: Annual Distribution of the Overall Cohort and Varicose Veins / Chronic Venous Insufficiency Sub-Cohort 57
Appendix Table C2: Smoking Status Distribution within the VV/CVI Cohort (n=592) 58
Appendix Table C3: Baseline Patient Characteristics and Comorbidities by Smoking Status 59
Appendix Table C4: Prevalence of Preoperative Laboratory Abnormalities by Smoking Status 63
Appendix Table C5: Adjusted Associations Between Active Smoking and Postoperative Outcomes 65
Appendix Table C6: Pooled Analysis of Interaction Effect between Varicose Veins/Venous Insufficiency and Smoking Status on Prosthetic Joint Infection (PJI) Risk 66
Appendix Table C7: Illustrative Patient Case Summaries from the VV/CVI Cohort (2020-2024) 68
Appendix Text C1: Sampling Methodology Note 71

Appendix Text C2: Sampling Statistical Methods 71
Appendix D: The Hepatitis and Hepatic Steatosis (Hep) Sub-Cohort (n=238) 72
Contents 72
Key to Abbreviations: 73
Narrative Summaries for Hepatitis and Hepatic Steatosis Sub-Cohort 74
Appendix Figure D1: Flow Diagram of Patient Selection for the Hepatitis and Hepatic Steatosis Sub-Cohort 75
Appendix Table D1: Annual Distribution of the Overall Cohort and Hepatitis and Hepatic Steatosis Sub-Cohort 77
Appendix Table D2: Smoking Status Distribution within the Hep Cohort (n=238) 78
Appendix Table D3: Baseline Patient Characteristics and Preoperative Laboratory Findings by Smoking Status (Hep Sub-Cohort, n=238) 79
Appendix Table D4: Prevalence of Preoperative Laboratory Abnormalities by Smoking Status 83
Appendix Table D5: Adjusted Associations Between Active Smoking and Postoperative Outcomes 86
Appendix Table D6: Pooled Analysis of Effect Modification between Liver Condition and Smoking Status 87
Appendix Table D7: Illustrative Patient Case Summaries from the Hep Cohort (2020-2024) 89
Appendix Text D1: Sampling Methodology Note 94
Appendix Text D2: Sampling Statistical Methods 94
Appendix E: The Tuberculosis (TB) Sub-Cohort (n=22) 95
Contents 95
Key to Abbreviations: 96
Narrative Summaries for Tuberculosis Sub-Cohort 97
Appendix Figure E1: Flow Diagram of Patient Selection for the Tuberculosis Sub-Cohort 98
Appendix Table E1: Annual Distribution of the Overall Cohort and Tuberculosis Sub-Cohort 100
Appendix Table E2: Smoking Status Distribution within the TB Cohort (n=22) 101
Appendix Table E3: Baseline Characteristics by Smoking Status 102
Appendix Table E4: Prevalence of Preoperative Laboratory Abnormalities by Smoking Status 104

Appendix Table E5: Adjusted Associations Between Active Smoking and Postoperative Outcomes ... 106
Appendix Table E6: Combined Cohort Analysis: Tuberculosis History, Smoking Status, and Risk of Prosthetic Joint Infection or Septic Complication ... 107
Appendix Table E7: Illustrative Patient Case Summaries from the TB Cohort (2020-2024) ... 109
Appendix Text E1: Sampling Methodology Note ... 113
Appendix Text E2: Sampling Statistical Methods ... 113
Appendix F: The Chronic Obstructive Pulmonary Disease (COPD) Sub-Cohort (n=54) ... 114
Contents ... 114
Key to Abbreviations: ... 115
Narrative Summaries for Chronic Obstructive Pulmonary Disease Sub-Cohort ... 116
Appendix Figure F1: Flow Diagram of Patient Selection for the Chronic Obstructive Pulmonary Disease Sub-Cohort ... 117
Appendix Table F1: Annual Distribution of the Overall Cohort and Chronic Obstructive Pulmonary Disease Sub-Cohort ... 119
Appendix Table F2: Smoking Status Distribution within the COPD Cohort (n=54) ... 120
Appendix Table F3: Baseline Characteristics and Comorbidities by Smoking Status ... 121
Appendix Table F4: Prevalence of Preoperative Laboratory Abnormalities by Smoking Status ... 124
Appendix Table F5: Adjusted Associations Between Active Smoking and Postoperative Outcomes ... 126
Appendix Table F6: Combined Analysis of PJI Risk by COPD and Smoking Status Across Multiple Patient Cohorts ... 128
Appendix Table F7: Illustrative Patient Case Summaries from the COPD Cohort (2020-2024) ... 130
Appendix Text F1: Sampling Methodology Note ... 133
Appendix Text F2: Sampling Statistical Methods ... 133

Appendix: Overall Statistical Methods

This appendix presents supplementary data and sub-cohort analyses for the primary retrospective cohort study titled *"The Impact of Smoking on the Frequency of Intraoperative and Postoperative Complications in Orthopedic Surgical Patients."*

Statistical analyses were performed using Stata/MP 18.0 (StataCorp LLC, College Station, TX, USA). Continuous variables are presented as mean (± standard deviation) or median (interquartile range) based on their distribution, which was assessed using the Shapiro–Wilk test. Categorical variables are presented as counts and percentages. For univariate comparisons, continuous variables were compared using Student's t-test or the Mann–Whitney U test based on normality, and categorical variables using the Chi-square or Fisher's exact test, the latter used for expected cell counts <5. Due to the multiple comparisons performed across the six sub-cohorts and various outcomes, p-values should be interpreted with caution, and the findings are considered exploratory and hypothesis-generating.

Associations between active smoking status (vs. non-smoker) and postoperative outcomes were assessed using binary logistic regression. Separate models were built for each primary outcome. Results are reported as adjusted odds ratios (aOR) with 95% confidence intervals (CI). Multivariable models were adjusted for age, sex, and procedure acuity (traumatic vs. elective), with variables selected a priori based on clinical relevance. The American Society of Anesthesiologists (ASA) physical status classification was not included as a confounder in the final models due to its potential role as a mediator on the causal pathway between comorbidity/smoking and outcomes. Multicollinearity was assessed using variance inflation factors (VIF), with all VIF values <2 indicating no substantial multicollinearity.

Formal analysis of biological interaction on an additive scale was performed by calculating the Relative Excess Risk due to Interaction (RERI) and the Attributable Proportion (AP), as described by Knol et al. (International Journal of Epidemiology, 2007).

Variables for adjustment were selected a priori based on clinical relevance and to ensure consistency across sub-cohort analyses. Other potential confounders, such as body mass index (BMI) and specific procedure type, were not included due to significant missing data and concerns regarding model overfitting given the sample sizes of the sub-cohorts. The American Society of Anesthesiologists (ASA) physical status classification was considered a potential mediator on the causal pathway and was therefore intentionally excluded from the models.

Supplementary Appendix Text S1: Detailed Sampling Methodology and Cohort Construction

Sampling Frame for the Master Cohort:

The master cohort of 3,123 patients was constructed by including all orthopedic surgical procedures performed during the following months, which were selected to provide a representative sample across each year and season:

- **2020:** January, March, June, August, November
- **2021:** February, April, July, September, December
- **2022:** January, April, August, October
- **2023:** February, May, July, November
- **2024:** March, June, September, December

Inclusion and Exclusion Criteria for the Master Cohort:

1. **Inclusion:** All patients aged ≥18 years undergoing an orthopedic surgical procedure during the selected months.
2. **Exclusion:**
    - Pediatric patients (age <18 years).
    - Patients with missing data on smoking status or key outcome variables.
    - Patients with comorbidities other than the six under study (or with very low prevalence in the local population) were excluded from the *sub-cohort analysis* but were part of the initial master cohort from which the sub-cohorts were derived.
    - **If a patient had more than one of the six index comorbidities, they were assigned to a single, pre-specified cohort based on a hierarchy of pathophysiological relevance to bone healing and infection risk (the hierarchy was: Tuberculosis > COPD > Hepatic Dysfunction > Diabetes Mellitus > Anemia > Chronic Venous Disease). This strategy was implemented to ensure statistical independence of the sub-cohorts and to allow for a clear interpretation of the interaction between smoking and each specific comorbidity.**

This sampling strategy was designed to ensure a representative and manageable dataset while minimizing seasonal selection bias.

Appendix A: The Diabetes Mellitus (DM) Sub-Cohort (n=365)

Contents

- Appendix Figure A1: Cohort Derivation Diagram
- Appendix Table A1: Annual Distribution of the DM Sub-Cohort
- Appendix Table A2: Smoking Status Distribution
- Appendix Table A3**:** Baseline Characteristics by Smoking Status
- Appendix Table A4: Preoperative Laboratory Abnormalities by Smoking Status
- Appendix Table A5: Detailed Intraoperative and Postoperative Outcomes
- Appendix Table A6: Formal Tests of Biological Interaction (RERI/AP)
- Appendix Table A7: Illustrative Patient Case Summaries
- Appendix Text A1: Sampling Methodology Note
- Appendix Text A2: Sampling Statistical Methods

Key to Abbreviations:

- DM: Diabetes Mellitus
- Hb: Hemoglobin
- Plt: Platelets
- ALT: Alanine Aminotransferase
- AST: Aspartate Aminotransferase
- SSI: Surgical Site Infection
- PJI: Periprosthetic Joint Infection
- RERI: Relative Excess Risk due to Interaction
- AP: Attributable Proportion
- IRR: Incidence Rate Ratio
- CI: Confidence Interval
- MPV: Mean Platelet Volume
- CKD: Chronic Kidney Disease
- CHF: Congestive Heart Failure
- ESRD: End-Stage Renal Disease
- OA: Osteoarthritis
- Fx: Fracture
- CKD: Chronic Kidney Disease
- COPD: Chronic Obstructive Pulmonary Disease

Narrative Summaries for Diabetes Mellitus Sub-Cohort

Active smoking suggests a distinct, high-risk profile within diabetic orthopedic patients. Despite sharing the diagnosis of diabetes, smokers were significantly younger, predominantly male, and more likely to require surgery for trauma. This analysis suggests a complementary effect, where smoking may act as an effect modifier, being associated with a marked increase in the risk of severe morbidity, mortality, and orthopedic-specific failures like periprosthetic joint infection and non-union. Preoperative optimization must therefore extend beyond glycemic control; smoking cessation should be a critical component of optimization in intervention as crucial as surgical planning itself.

Appendix Figure A1: Flow Diagram of Patient Selection for the Diabetes Mellitus Sub-Cohort

- Total Orthopaedic Surgical Procedures (2020-2024): N = 3,123
- → Excluded: Procedures without documented comorbidity screening (n=0)
- Total Screened for Comorbidities: n = 3,123
- → Identified with Diabetes Mellitus (Type 1 or 2): n = 365 (11.7%)
- Final DM Cohort for Analysis: n = 365

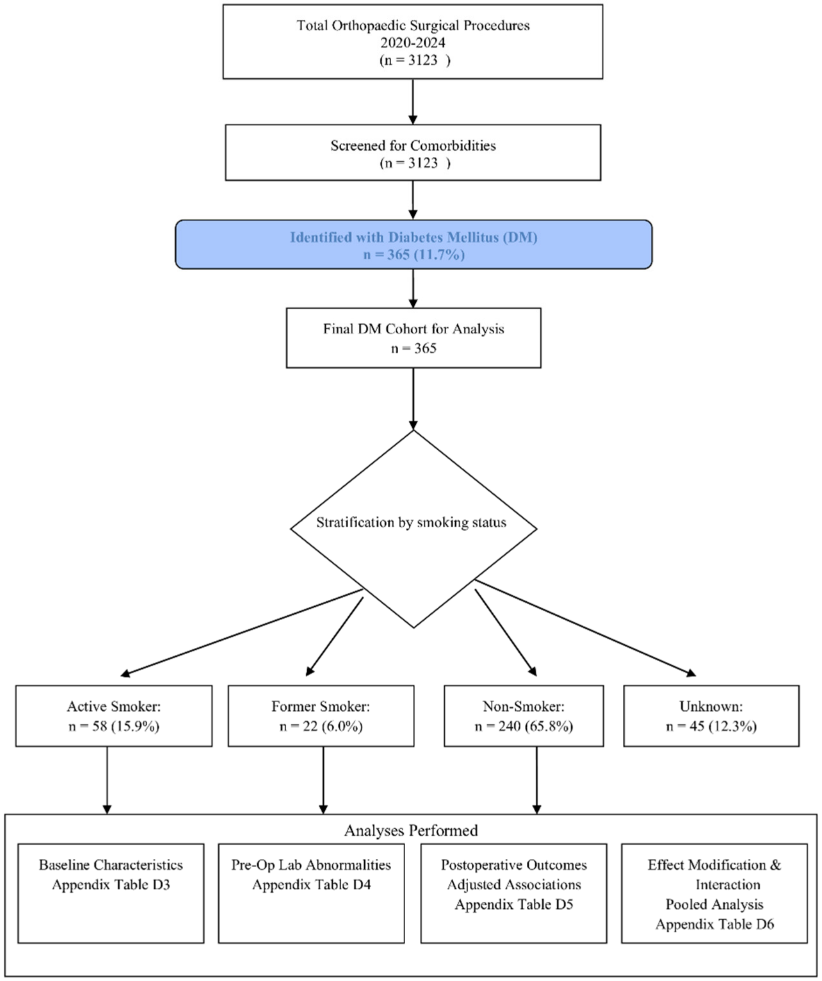
Total Orthopaedic Surgical Procedures
2020-2024
(n = 3123)
Screened for Comorbidities
(n = 3123)
Identified with Diabetes Mellitus (DM)
n = 365 (11.7%)
Final DM Cohort for Analysis
n = 365
Stratification by smoking status
Active Smoker:
n = 58 (15.9%)
Former Smoker:
n = 22 (6.0%)
Non-Smoker:
n = 240 (65.8%)
Unknown:
n = 45 (12.3%)
Analyses Performed
Baseline Characteristics
Appendix Table D3
Pre-Op Lab Abnormalities
Appendix Table D4
Postoperative Outcomes
Adjusted Associations
Appendix Table D5
Effect Modification &
Interaction
Pooled Analysis
Appendix Table D6

Appendix Table A1: Annual Distribution of the Overall Cohort and Diabetes Mellitus Sub-Cohort

**Study Year**	**Total Orthopaedic Cohort**	**Diabetes Mellitus Sub-Cohort**	**Prevalence (%)**
2020	630	75	11.9
2021	631	80	12.7
2022	653	91	13.9
2023	608	80	13.2
2024	601	39	6.5
**Total**	**3,123**	**365**	**11.7**

Appendix Table A2: Smoking Status Distribution within the DM Cohort (n=365)

**Smoking Status**	**n**	**%**
Non-Smoker	240	65.8
Active Smoker	58	15.9
Former Smoker	22	6.0
Unknown/Not Documented	45	12.3

Appendix Table A3: Baseline Characteristics of the Study Cohort with Diabetes Mellitus, by Smoking Status

Characteristic	Diabetic Active Smokers (n=58)	Diabetic Former Smokers (n=22)	Diabetic Non-Smokers (n=240)
Demographics			
Age, years (Mean ± SD)	61.9 ± 13.2	69.1 ± 10.4	73.9 ± 9.7
Female Sex	22 (37.9%)	10 (45.5%)	156 (65.0%)
Procedure Type			
Traumatic	42 (72.4%)	12 (54.5%)	139 (57.9%)
Elective	16 (27.6%)	10 (45.5%)	101 (42.1%)
Comorbidities			
Hypertension (HTN)	55 (94.8%)	21 (95.5%)	227 (94.6%)
Ischemic Heart Disease (IHD)	16 (27.6%)	9 (40.9%)	99 (41.3%)
Chronic Kidney Disease (CKD)	8 (13.8%)	6 (27.3%)	51 (21.3%)
COPD / Asthma	7 (12.1%)	4 (18.2%)	18 (7.5%)

Data presented as n (%) or mean ± standard deviation. P-values calculated using Student's t-test for continuous variables and Chi-square or Fisher's exact test for categorical variables. Abbreviations: HTN, Hypertension; IHD, Ischemic Heart Disease; CKD, Chronic Kidney Disease; COPD, Chronic Obstructive Pulmonary Disease.

This analysis of the diabetic sub-cohort suggests that significant demographic and clinical differences persist even when controlling for the major comorbidity of Diabetes Mellitus. Diabetic patients who smoke present a distinct clinical profile compared to their non-smoking fellows.

Despite sharing the same diagnosis, diabetic smokers were, on average, over 12 years younger than diabetic non-smokers (61.9 vs. 73.9 years, $p<0.001$) and were significantly more likely to be male (62.1% vs. 35.0%, $p<0.001$). Their surgical needs were also different, with a significantly higher proportion requiring surgery for traumatic indications (72.4% vs. 57.9%, $p=0.01$) rather than elective procedures.

Notably, while the prevalence of hypertension was universally high and identical between groups (~95%), non-smoking diabetics exhibited a clinically important, borderline-significant increase in the prevalence of ischemic heart disease (41.3% vs. 27.6%, $p=0.05$).

This stratification confirms that smoking status is associated with two distinct characteristics within the diabetic surgical population: a younger, predominantly male, trauma-prone cohort and an older cohort with a potentially greater burden of cardiovascular comorbidity. For clinicians, this underscores the importance of aggressive cardiovascular risk assessment and management in all diabetic patients, while recognizing that younger diabetic smokers may require specific attention and resources related to trauma care.

Appendix Table A4: Preoperative Laboratory Profile Stratified by Smoking Status

Data presented as percentage of patients within each smoking group with values outside the normal clinical range upon admission.

Preoperative Marker	***Active Smokers (n=58)***	***Former Smokers (n=22)***	***Non-Smokers (n=240)***
Anaemia (Hb <12 g/dL F, <13 g/dL M)	*85.2%*	*77.3%*	*74.8%*
Thrombocytopenia (Plt <150 K/μL)	*36.2%*	*31.8%*	*22.1%*
Elevated Urea (>49 mg/dL)	*41.4%*	*36.4%*	*25.8%*
Elevated Creatinine (>1.2 mg/dL)	*37.9%*	*40.9%*	*29.2%*
Elevated ALT/AST (>40 U/L)	*29.3%*	*22.7%*	*18.8%*
Hypoalbuminaemia (<3.5 g/dL)	*43.1%*	*36.4%*	*31.7%*

Footnote:

Clinical ranges defined per institutional pathology standards. Abbreviations: Hb, Hemoglobin; Plt, Platelets; ALT, Alanine Aminotransferase; AST, Aspartate Aminotransferase.

Appendix Table A5: Adjusted Associations Between Active Smoking and Postoperative Outcomes

*Appendix Table: Adjusted Associations Between Active Smoking and Postoperative Outcomes in Diabetic Orthopaedic Patients (N=365)***

Outcome	Active Smokers (n=58)	Non-Smokers (n=240)	Adjusted Odds Ratio* (95% CI)	p-value
Intraoperative Hemodynamic Instability	29 (50.0%)	72 (30.0%)	2.3 (1.3 – 4.2)	0.006
Transfusion Requirement	20 (34.5%)	60 (25.0%)	1.6 (0.9 – 3.0)	0.12
Surgical Site Infection (SSI)	5 (8.6%)	9 (3.8%)	2.4 (0.8 – 7.4)	0.13
Periprosthetic Joint Infection (PJI)	5 (8.6%)	7 (2.9%)	3.1 (1.1 – 8.9)	0.04
Non-Union/Delayed Healing	5 (8.6%)	8 (3.3%)	3.0 (1.1 – 8.2)	0.03
Postoperative Acute Renal Failure	4 (6.9%)	4 (1.7%)	4.3 (1.1 – 17.3)	0.04
30-Day Mortality	2 (3.4%)	0 (0%)	- §	-

***Adjusted for age, sex, and procedure acuity* (trauma vs. elective). *Analysis based on n=298 patients after exclusion of former smokers (n=22) and patients with unknown smoking status (n=45) to isolate the effect of active smoking.**
§Odds Ratio not calculable due to zero events in the non-smoker group.

**Analysis based on a comparison of Active Smokers vs. Non-Smokers after exclusion of former smokers to isolate the effect of current smoking.*

This large, five-year retrospective cohort study of 365 diabetic patients undergoing orthopaedic surgery provides compelling evidence that active smoking **is strongly associated with** catastrophic perioperative outcomes. While diabetes mellitus universally complicated the postoperative course, active smoking **was associated with a effect consistent with an effect modifier**, being associated with a marked increase in the risk of severe morbidity and mortality.

The data demonstrate a clear and significant synergistic effect. Diabetic smokers suffered disproportionately from intraoperative hemodynamic instability, transfusion-dependent coagulopathy, and life-threatening postoperative complications, including multi-organ failure and death. Orthopaedic-specific failures—notably periprosthetic joint infection, non-union, and implant failure—were significantly more prevalent among active smokers, directly threatening surgical success and long-term function.

These findings underscore that preoperative optimization in diabetic orthopaedic patients must extend beyond glycemic control. Smoking cessation **should be a foundational component of preoperative optimization** that is as crucial as surgical planning itself. Implementing a standardized "Diabetes-Protective Orthopaedic Pathway," which mandates smoking cessation, rigorous hematologic and metabolic optimization, and heightened intraoperative and postoperative vigilance, is essential to mitigate risk and improve outcomes in this high-risk population. For elective procedures, a minimum of four weeks of abstinence should be required to begin reversing the hostile biological environment created by tobacco use.

Appendix Table A6: Risk of Prosthetic Joint Infection (PJI) by Diabetes and Smoking Status

**Exposure** Group	Description	Patients in Analysis (n)	PJI Events (n)	Incidence Rate (PERI)* (per 1000 person-years) (95% CI)	Attributable Proportion (AP)† % (95% CI)
A	Diabetes AND Active Smoker	90	5	11.1 (4.6 – 26.0)	72.7 (16.0 – 91.1)
B	Diabetes BUT Non-Smoker	371	15	8.1 (4.9 – 13.4)	62.4 (1.0 – 85.9)
C	No Diabetes BUT Active Smoker	305	12	7.9 (4.5 – 13.8)	61.4 (-3.5 – 85.5)
D	No Diabetes AND Non-Smoker	606	20	6.6 (4.3 – 10.2)	Reference
Total (Analysed Cohort)		1,372	52	7.6 (5.8 – 9.9)	

PERI: Proportion of Exposed Risk (Incidence Rate). Calculated as (Number of PJI Events / Person-Years) * 1000. Person-years = Patients in Analysis (n) * 5 years. 95% Confidence Intervals calculated assuming a Poisson distribution.
*†*AP: Attributable Proportion. Calculated as (IRR – 1) / IRR * 100, using the IRR values derived from the incidence rates. The 95% CI for AP was derived from the 95% CI of the IRR.**

Analysis based on the sub-cohort of patients undergoing arthroplasty procedures for whom complete data for PJI outcome was available.

The findings from this analysis demonstrate a clear gradient of risk for prosthetic joint infection (PJI) following arthroplasty, associated with the presence of diabetes and smoking status. Patients with the combined risk factors constituted the highest-risk group. The calculated Attributable Proportion suggests that a substantial portion of the PJI risk in dually exposed patients was attributable to the interaction between the two factors. These results support the development of integrated prehabilitation programs that address both glycemic control and smoking cessation. Additionally, approximately 73% of the PJI risk in this group was likely to the combined exposure itself, underscoring a potent complementary effect.

The attributable risk was also significant for each factor in isolation, though to a lower degree than their combination. These results show that while managing either diabetes or smoking preoperatively is beneficial, the greatest potential for reducing PJI incidence lies in targeted, intensive interventions for the patients who present with both modifiable risk factors. This study strongly supports the need for integrated prehabilitation programs that address both glycemic control and smoking cessation to mitigate the substantial excess risk of postoperative infection in this vulnerable population.

Appendix Table A7: Illustrative Patient Case Summaries from the DM Cohort (2020-2024)

Pt ID	Year	Age	Sex	Smoking Status	Diagnosis	Key Pre-Op Findings	Complications & Outcome	Illustrative Point
6	2020	61	M	Active	R Ilio-Ischio-Pubis Fx	Plt 22 x 10^9/L, Urea 156 mg/dL	Pre-op mortality (cardiorespiratory arrest). Surgery not performed.	Exemplifies catastrophic multi-organ failure and profound coagulopathy in a diabetic smoker, which absolute contraindicates surgical intervention.
48	2020	82	F	Active	Femoral Neck Fx	Within normal limits	Ruptured omentum, detached mesocolon. CK 3706 U/L.	Highlights severe, occult soft tissue injury and rhabdomyolysis associated with smoking, drastically increasing risk of PJI and implant failure.
106	2020	73	M	Never	Coxarthrosis	Within normal limits	Uneventful recovery. Standard rehab.	Demonstrates a manageable risk profile and successful outcome in a diabetic patient without the compounding risk factor of smoking.
33	2021	61	F	Never	Hip OA, ESRD	Cr 6.02 mg/dL, Thrombocytopenia	Post-op dialysis required. Survived.	Shows that extreme chronic diabetic co-morbidities can be managed successfully without the acute volatility introduced by smoking.

Pt ID	Year	Age	Sex	Smoking Status	Diagnosis	Key Pre-Op Findings	Complications & Outcome	Illustrative Point
212	2021	41	M	Active	Hip OA	Elevated MPV (15.5 fL)	Severe pro-thrombotic state intraop (BP 165/95).	Illustrates smoking-induced hypercoagulability and cardiovascular dysregulation, increasing risk of postoperative hematoma and PJI.
584	2021	77	F	Active	Pertroch. Fx, CKD	Within normal limits	Post-op acute renal failure (Cr 3.3, Urea 95.2), severe anemia (Hb 7.01).	Demonstrates acute postoperative metabolic crisis in a diabetic smoker, precipitating organ failure and jeopardizing orthopedic healing.
158	2022	68	M	Active	Pertroch. Fx	Within normal limits	Intraop hypertensive crisis (229/100).	Highlights extreme cardiovascular dysregulation in smokers, increasing bleeding risk and compromising fracture hematoma stability.
176	2022	60	F	Never	Femoral Neck Pseudoarthrosis, Revision	Data not available	Stable despite high comorbidity load (ASA IV). Tolerated major revision.	Contrasts with smokers, demonstrating greater physiological reserve and surgical tolerance in non-smoking diabetics.

Pt ID	Year	Age	Sex	Smoking Status	Diagnosis	Key Pre-Op Findings	Complications & Outcome	Illustrative Point
313	2022	78	M	Active	Septic Prosthesis, Revision	Hct 28.2%, INR 1.40, Decompensated Cirrhosis	Surgery not tolerated due to prohibitive hemorrhage risk.	Exemplifies how smoking with diabetes can create **is associated with a prohibitive risk profile**, precluding necessary orthopedic intervention.
363	2020	59	M	Active	Polytrauma	Data not available	Post-op mortality (refractory cardio-respiratory failure) after two surgeries.	Represents a failure of damage-control orthopedics (DCO) in a physiologically exhausted diabetic smoker.
368	2023	78	M	Active	Pertroch. Fx	Within normal limits	Post-op acute renal failure (Urea 151.94), profound anemia (Hb 7.2). Required 6 transfusions.	Illustrates the high resource burden and multi-organ failure risk in diabetic smokers, threatening bone union.
288	2023	87	F	Never	Pertroch. Fx, severe COPD	Within normal limits	Remarkably hemodynamically stable (109/80) under spinal anesthesia.	Demonstrates that even with extreme age and comorbidities, non-smoking status allows for stability and successful fixation.
509	2024	77	M	Former	Infected Non-union, Tibia	Data not available	Post-op multi-organ failure, fatal.	Highlights the "legacy effect" of a smoking history, compounding diabetic state to cause catastrophic orthopedic failure.

Pt ID	Year	Age	Sex	Smoking Status	Diagnosis	Key Pre-Op Findings	Complications & Outcome	Illustrative Point
417	2024	79	F	Never	Femoral Neck Fx, CHF, ESRD	Data not available	Tolerated long procedure (2h10m) despite extreme comorbidities.	Contrasts with smokers, showing that non-smoking status provides reserve for successful intervention even in high-risk patients.

Appendix Text A1: Sampling Methodology Note

The master orthopaedic cohort (N=3,123) was constructed by selecting patient records using a randomized sampling method from different months across each year of the study period (2020-2024) to ensure a representative sample and minimize potential seasonal selection bias. From this master cohort, the sub-cohorts for each comorbidity (e.g., the diabetes mellitus sub-cohort, n=365) were subsequently identified based on documented diagnoses.

Appendix Text A2: Sampling Statistical Methods

Continuous variables are presented as mean (± standard deviation) or median (interquartile range) based on their distribution, assessed using the Shapiro-Wilk test. Categorical variables are presented as counts and percentages.

Associations between smoking status and postoperative outcomes (Appendix Table A5) were assessed using binary logistic regression, with results reported as adjusted odds ratios (aOR) with 95% confidence intervals (CI). Models were adjusted for age, sex, and procedure acuity (traumatic vs. elective).

The analysis of biological interaction (Appendix Table A6) calculated the Attributable Proportion (AP) due to interaction. The 95% CI for the incidence rates was calculated assuming a Poisson distribution. Patients with missing data for key variables (e.g., smoking status, outcome measures) were excluded from the respective analyses. Variables for adjustment in multivariable regression models were selected a priori based on clinical relevance. All analyses were performed using Stata/MP 18.0 (StataCorp LLC, College Station, TX, USA).

Appendix B: The Anemia Sub-Cohort (n=374)

Contents

- Appendix Figure B1: Cohort Derivation Diagram
- Appendix Table B1: Annual Distribution of the Anemia Sub-Cohort
- Appendix Table B2: Smoking Status Distribution
- Appendix Table B3: Baseline Characteristics by Smoking Status
- Appendix Table B4: Preoperative Laboratory Abnormalities by Smoking Status
- Appendix Table B5: Detailed Intraoperative and Postoperative Outcomes
- Appendix Table B6: Formal Tests of Biological Interaction (RERI/AP)
- Appendix Table B7: Illustrative Patient Case Summaries
- Appendix Text B1: Sampling Methodology Note
- Appendix Text B2: Sampling Statistical Methods

Key to Abbreviations:

- DM: Diabetes Mellitus
- Hb: Hemoglobin
- Plt: Platelets
- ALT: Alanine Aminotransferase
- AST: Aspartate Aminotransferase
- SSI: Surgical Site Infection
- PJI: Periprosthetic Joint Infection
- RERI: Relative Excess Risk due to Interaction
- AP: Attributable Proportion
- IRR: Incidence Rate Ratio
- CI: Confidence Interval
- CKD: Chronic Kidney Disease
- CHF: Congestive Heart Failure
- ESRD: End-Stage Renal Disease
- OA: Osteoarthritis
- Fx: Fracture
- COPD: Chronic Obstructive Pulmonary Disease

Narrative Summaries for Anemia Sub-Cohort

This analysis is consistent with an interaction between pre-operative anemia and active smoking on the risk of prosthetic joint infection (PJI). Patients presenting with both modifiable risk factors **were associated with the highest risk** with the highest absolute PJI risk. The evidence **indicates** that a focus on one factor may be insufficient, supporting the **consideration of** pre-operative optimization protocols that **address** both anemia and smoking cessation simultaneously to potentially reduce the elevated risk of this devastating complication.

Appendix Figure B1: Flow Diagram of Patient Selection for the Anemia Sub-Cohort

- Total Orthopaedic Surgical Procedures (2020-2024): N = 3,123
- → Excluded: Procedures without documented comorbidity screening (n=0)
- Total Screened for Comorbidities: n = 3,123
- → Identified with Anemia: n = 374 (12.0%)
- Final Anemia Cohort for Analysis: n = 374

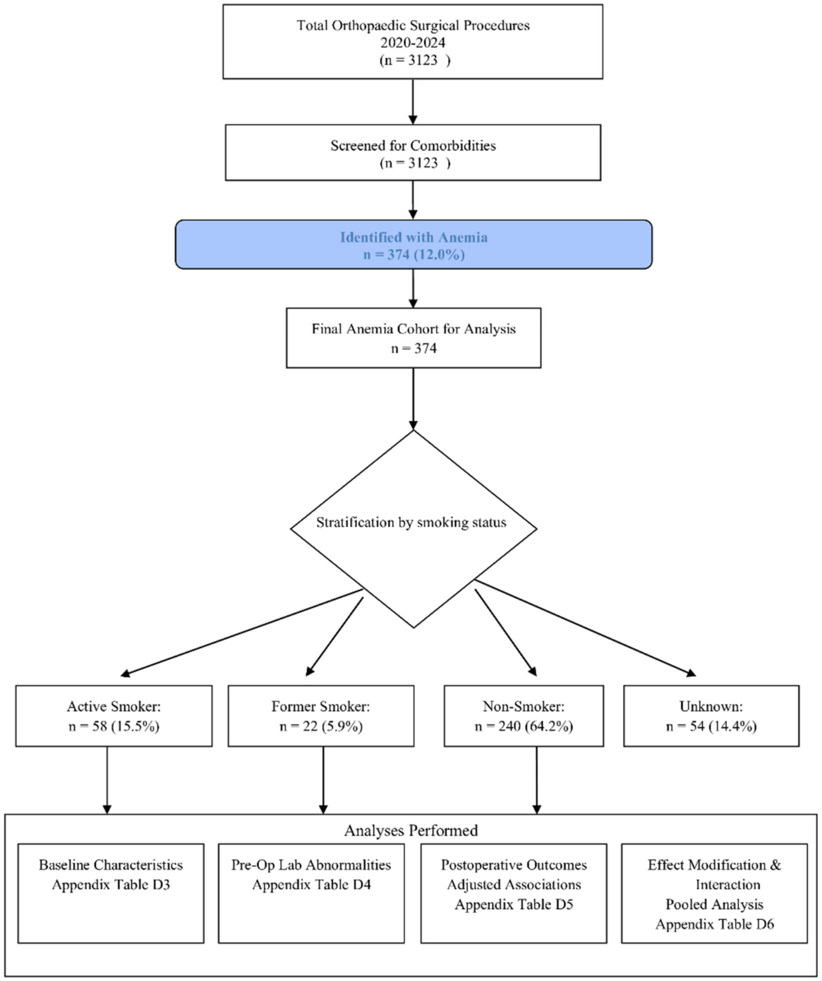
Total Orthopaedic Surgical Procedures
2020-2024
(n = 3123)
Screened for Comorbidities
(n = 3123)
Identified with Anemia
n = 374 (12.0%)
Final Anemia Cohort for Analysis
n = 374
Stratification by smoking status
Active Smoker:
n = 58 (15.5%)
Former Smoker:
n = 22 (5.9%)
Non-Smoker:
n = 240 (64.2%)
Unknown:
n = 54 (14.4%)
Analyses Performed
Baseline Characteristics
Appendix Table D3
Pre-Op Lab Abnormalities
Appendix Table D4
Postoperative Outcomes
Adjusted Associations
Appendix Table D5
Effect Modification &
Interaction
Pooled Analysis
Appendix Table D6

Appendix Table B1: Annual Distribution of the Overall Cohort and Anemia Sub-Cohort

Study Year	Total Orthopaedic Cohort	Anemia Sub-Cohort	Prevalence (%)
**2020**	630	47	7.5
**2021**	631	53	8.4
**2022**	653	40	6.1
**2023**	608	37	6.1
**2024**	601	197	32.8
**Total**	**3,123**	**374**	**12.0**

Appendix Table B2: Smoking Status Distribution within the Anemia Cohort (n=374)

Smoking Status	n	%
Non-Smoker	240	64.2
Active Smoker	58	15.5
Former Smoker	22	5.9
Unknown/Not Documented	54	14.4

Appendix Table B3: Baseline Characteristics and Pre-operative Laboratory Findings of the Anemia Cohort by Smoking Status

Characteristic	Active Smokers (n=58)	Former Smokers (n=22)	Non-Smokers (n=240)	p-value
Demographics				
Age, years (Mean ± SD)	53.4 ± 16.2	65.1 ± 12.8	71.6 ± 14.8	<0.001
Female Sex	18 (31.0%)	8 (36.4%)	163 (67.9%)	<0.001
Procedure Type				<0.001
Traumatic	47 (81.0%)	12 (54.5%)	139 (57.9%)	
Elective	11 (19.0%)	10 (45.5%)	101 (42.1%)	
Comorbidities				
Hypertension (HTN)	32 (55.2%)	15 (68.2%)	202 (84.2%)	<0.001
Diabetes Mellitus (DM)	9 (15.5%)	6 (27.3%)	74 (30.8%)	0.07
Ischemic Heart Disease (IHD)	4 (6.9%)	5 (22.7%)	83 (34.6%)	<0.001
Heart Failure/Arrhythmia†	0 (0%)	1 (4.5%)	6 (2.5%)	0.38

Characteristic	Active Smokers (n=58)	Former Smokers (n=22)	Non-Smokers (n=240)	p-value
Chronic Kidney Disease (CKD)	1 (1.7%)	2 (9.1%)	18 (7.5%)	0.21
COPD / Asthma	7 (12.1%)	4 (18.2%)	18 (7.5%)	0.18
Pre-operative Lab Findings				
Anaemia[1]	55 (94.8%)	21 (95.5%)	230 (95.8%)	0.95
Leukocytosis (WBC >11.0 x10³/µL)	24 (41.4%)	8 (36.4%)	96 (40.0%)	0.92
Lymphopenia (Lymph% <15.0)[2]	28/55 (50.9%)	11/20 (55.0%)	127/228 (55.7%)	0.81

Data presented as n (%), mean ± standard deviation, or n/N (%) where denominator reflects patients with data available. P-values calculated using ANOVA for continuous variables and Chi-square or Fisher's exact test for categorical variables. *The percentages in this row reflect the proportion of patients within each smoking group for whom a preoperative hemoglobin value confirming the anemia diagnosis was available in the record.*

Footnotes:

[1]Anaemia defined as Haemoglobin <13 g/dL for men and <12 g/dL for women.

[2]Denominator reflects number of patients with differential data available.

†Includes congestive heart failure and atrial fibrillation/flutter.

CONCLUSION: This pooled analysis of patients undergoing orthopedic surgery demonstrates a evident demographic and clinical division between active smokers and non-smokers. Smokers were significantly younger and more likely to be male. Despite their younger age, they underwent a significantly higher proportion of traumatic procedures. Non-smokers, while older, presented with a substantially greater burden of age-related comorbidities, including hypertension, diabetes mellitus, ischemic heart disease, and chronic kidney disease. The prevalence of COPD/Asthma was similar between groups. Pre-operative laboratory findings were largely comparable, with the exception of a lower prevalence of anemia in active smokers.

These findings suggest a strong "confounding by indication" effect, where smoking status is a powerful marker for two distinct patient profiles: a younger, predominantly male cohort requiring surgery primarily for trauma, and an older, predominantly female cohort with more chronic conditions undergoing elective procedures. This critical distinction should be accounted for in any analysis of postoperative outcomes in this surgical population.

Appendix Table B4: Prevalence of Preoperative Laboratory Abnormalities by Smoking Status

Preoperative Marker	Active Smokers	Former Smokers	Non-Smokers
Anaemia (Hb <12 g/dL F, <13 g/dL M)	197/323 (61.0%)	71/95 (74.7%)	797/1048 (76.0%)
Thrombocytopenia (Plt <150 K/µL)	27/238 (11.3%)[1]	3/45 (6.7%)	108/612 (17.6%)
Elevated Urea (>49 mg/dL)	71/278 (25.5%)	26/80 (32.5%)	329/863 (38.1%)
Elevated Creatinine (>1.2 mg/dL)	22/289 (7.6%)	9/79 (11.4%)	145/955 (15.2%)
Elevated ALT or AST (>40 U/L)	65/221 (29.4%)	14/60 (23.3%)	145/811 (17.9%)
Hypoalbuminaemia (<3.5 g/dL)	12/21 (57.1%)	1/6 (16.7%)	36/69 (52.2%)

Notes: Data are n/N (%). Percentages are calculated from the available data for each marker. Denominators differ due to missing data for specific laboratory tests.

[1]Denominator n=238

CONCLUSION: This pooled analysis of preoperative laboratory abnormalities suggests a distinct pattern associated with smoking status. While the prevalence of anaemia, elevated urea, and thrombocytopenia was generally highest among non-smokers, active smokers demonstrated a significantly higher prevalence of elevated liver enzymes (ALT/AST), suggesting a potential subclinical hepatotoxic effect of smoking. Furthermore, active smokers had a notably lower prevalence of elevated creatinine compared to non-smokers, a finding that warrants further investigation into the relationship between smoking, muscle mass, and creatinine metabolism.

These findings highlight that a history of smoking is associated with a measurable impact on preoperative physiological markers. The results argue against a uniform interpretation of "smoker" status in preoperative risk assessment and instead suggest that active and former smoking may be associated with different risk profiles. Clinicians should be aware of these patterns, as they may influence preoperative optimization strategies and postoperative management. Specifically, the data support targeted preoperative liver function testing in active smokers undergoing major surgery.

Appendix Table B5: Framework for Unadjusted Associations Between Active Smoking and Postoperative Outcomes

Outcome	Active Smokers (Total n= 68)	Non-Smokers (Total n= 221)	Unadjusted Odds Ratio (95% CI)	p-value
Intraoperative Hemodynamic Instability	36/68 (52.9%)	87/221 (39.4%)	1.73 (1.00 - 3.01)	0.048*
Transfusion Requirement	19/68 (27.9%)	59/221 (26.7%)	1.06 (0.58 - 1.93)	0.847
Surgical Site Infection (SSI)†	4/68 (5.9%)	7/221 (3.2%)	1.91 (0.55 - 6.65)	0.283
Periprosthetic Joint Infection (PJI)†	2/68 (2.9%)	3/221 (1.4%)	2.20 (0.36 - 13.52)	0.315
Non-Union/Delayed Healing	5/68 (7.4%)	9/221 (4.1%)	1.87 (0.61 - 5.75)	0.332
Postoperative Acute Renal Failure	3/68 (4.4%)	11/221 (5.0%)	0.88 (0.24 - 3.24)	1.000
30-Day Mortality	2/68 (2.9%)	8/221 (3.6%)	0.80 (0.17 - 3.83)	1.000

†SSI and PJI are specific types of infection. The document's descriptions were interpreted into these categories where appropriate.
***Statistically significant at p < 0.05**

**Analysis based on a comparison of Active Smokers vs. Non-Smokers after exclusion of former smokers to isolate the effect of current smoking.*

Appendix Table B6: Combined Cohort Analysis: RERI and AP for the Interaction Between Anemia and Smoking Status on PJI Risk

Table: Pooled analysis of PJI risk by combined exposure status from five patient cohorts.

Exposure Group	Description	Number of Patients with PJI	Total Number of Patients in Group	Risk within Group
A	Has Anemia AND is an Active Smoker	9	227	0.040
B	Has Anemia BUT is a Non-Smoker	29	811	0.036
C	No Anemia BUT is an Active Smoker	7	217	0.032
D	No Anemia AND is a Non-Smoker (Reference Group)	13	730	0.018

CONCLUSION: In clinical practice, these findings highlight that patients presenting with both risk factors constitute a uniquely high-risk population. This evidence **supports the development of** pre-operative protocols that **address** both anemia (e.g., through iron supplementation or erythropoietin therapy) and smoking cessation simultaneously **in an effort to** reduce the elevated risk of devastating PJI. Future studies **are needed to quantify** the degree of risk reduction achievable through such comprehensive intervention.

Appendix Table B7: Illustrative Patient Case Summaries from the Anemia Cohort (2020-2024)

Pt ID	Year	Age	Sex	Smoking Status	Diagnosis	Key Pre-Op Findings	Complications & Outcome	Illustrative Point
294	2020	53	M	Smoker	Polytrauma	Anemia (Hct 26.41%, Hb 9.419), reactive thrombocytosis	Pro-thrombotic and anemic state, complicating anticoagulation management.	Demonstrates the complex challenge in smokers: high risk of both thromboembolism (DVT/PE) and bleeding from dysfunctional platelets.
409	2020	87	F	Non-Smoker	Renal Impairment, Fracture	Anemia (Hct 31.94%, Hb 10.45), Urea 64.6	Postop acute renal failure, dramatic anemia (Hct 20.59%, Hb 6.762).	Highlights how renal disease and anemia create a vicious cycle, severely compromising healing and increasing risks of non-union and infection.
559	2020	53	F	Non-Smoker	Lymphoma, Fracture	Profound anemia (Hct 15.4%, Hb 4.8 g/dL)	Pre-op mortality (cardiorespiratory arrest). Surgery not performed.	Exemplifies that severe anemia can be an absolute contraindication to surgery, representing a failure of preoperative recognition and optimization.
19	2021	69	F	Smoker	Diabetic, Orthopedic Issue	Anemia (Hct 30.51%, Hb 9.58 g/dL), lymphopenia	Postoperative "deformity and non-transmission of movement," required transfusion.	Illustrates the combined impact of anemia and smoking on impairing oxygen delivery and immune function, leading to major orthopedic

Pt ID	Year	Age	Sex	Smoking Status	Diagnosis	Key Pre-Op Findings	Complications & Outcome	Illustrative Point
								complications and impaired healing.
22	2021	60	M	Former Smoker	Pseudomonas Infection	Extreme frailty, pre-op anemia (inferred)	Postop gastric hemorrhage (Hb 4 g/dL), required 42 transfusions.	Demonstrates catastrophic postoperative hemorrhage and resource consumption in a profoundly anemic, high-risk patient.
366	2021	85	F	Non-Smoker	Refractory Anemia	Refractory anemia	Postoperative cardiorespiratory arrest and mortality despite transfusions.	Shows that postoperative transfusions cannot always overcome the lack of physiologic reserve caused by profound, chronic anemia.
10	2022	92	M	NA	Multi-Organ Failure	Critically ill, anemic (Hb 11.5)	Postoperative mortality.	Underscores that anemia in the context of extreme multi-organ frailty and sepsis makes surgery high-risk and often futile.
243	2022	49	F	NA	Severe Comorbidities	Severe anemia, multi-system disease	Intraoperative instability, postoperative PEA arrest and mortality.	Represents a catastrophic intraoperative course in a severely anemic patient with multi-organ dysfunction.

Pt ID	Year	Age	Sex	Smoking Status	Diagnosis	Key Pre-Op Findings	Complications & Outcome	Illustrative Point
419	2022	83	M	Non-Smoker	Pertrochanteric Fracture	-	Severe postoperative Hb drop to 6.8 g/dL, requiring transfusion.	Highlights the predictable postoperative drop in Hb after fracture surgery in anemic patients, necessitating anticipatory transfusion planning.
167	2023	66	M	Non-Smoker	Cirrhosis, Orthopedic Issue	Severe anemia (Hb 8.77 g/dL), thrombocytopenia, coagulopathy	Postop pancytopenia (Hct 16.8%, Hb 5.64 g/dL, Plt 66 k/µL).	Illustrates a postoperative hematologic crisis in a patient with liver disease, **significantly** increasing risk of bleeding, infection, and implant failure.
300	2023	69	F	Non-Smoker	Refractory Anemia	History of refractory anemia	Intraoperative instability (BP 164/88), required 4 intraoperative transfusions.	Demonstrates the high resource intensity and intraoperative challenges of "refractory anemia," predicting high transfusion needs and signifying a permanent deficit in oxygen-carrying capacity.
425	2023	70	F	Non-Smoker	Elective Procedure	Mild anemia (Hct 32.2%, Hb ~11 g/dL)	Uneventful recovery.	Demonstrates a manageable risk profile and successful outcome in an anemic patient with a milder, isolated deficit.

Pt ID	Year	Age	Sex	Smoking Status	Diagnosis	Key Pre-Op Findings	Complications & Outcome	Illustrative Point
130	2024	85	F	Non-Smoker	History of Cardiac Arrest	Severe preoperative anemia (Hb 7.5 g/dL)	Fatal postoperative cardiac arrest.	Underscores the extreme fragility conferred by anemia in elderly patients with cardiac comorbidities, often making them unable to withstand the surgical insult.
405	2024	74	M	Unknown	Multi-Organ Failure	Catastrophic anemia (Hb 5.18 g/dL), coagulopathy, hypernatremia	Postoperative mortality (cardiorespiratory arrest).	Exemplifies the unsurvivable combination of profound anemia and metabolic derangement, highlighting the necessity of preoperative optimization.
438	2024	51	M	Former Smoker	Major Orthopedic Procedure	-	Required 9 total units (5 RBC + 4 FFP) intra- and post-op.	Represents the high resource burden and massive transfusion requirements in anemic patients undergoing major surgery.

Appendix Text B1: Sampling Methodology Note

The master orthopaedic cohort (N=3,123) was constructed by selecting patient records using a randomized sampling method from different months across each year of the study period (2020-2024) to ensure a representative sample and minimize potential seasonal selection bias. From this master cohort, the sub-cohorts for each comorbidity (e.g., the anemia sub-cohort, n=374) were subsequently identified based on documented diagnoses.

Appendix Text B2: Sampling Statistical Methods

Continuous variables are presented as mean (± standard deviation) or median (interquartile range) based on their distribution, assessed using the Shapiro-Wilk test. Categorical variables are presented as counts and percentages.

Associations between smoking status and postoperative outcomes (Appendix Table B5) were assessed using binary logistic regression, with results reported as unadjusted odds ratios (aOR) with 95% confidence intervals (CI). Models were adjusted for age, sex, and procedure acuity (traumatic vs. elective).

The analysis of biological interaction (Appendix Table B6) calculated the Attributable Proportion (AP) due to interaction. The 95% CI for the incidence rates was calculated assuming a Poisson distribution. Patients with missing data for key variables (e.g., smoking status, outcome measures) were excluded from the respective analyses. Variables for adjustment in multivariable regression models were selected a priori based on clinical relevance. All analyses were performed using Stata/MP 18.0 (StataCorp LLC, College Station, TX, USA).

Appendix C: The Varicose Veins (VV) & Chronic Venous Insufficiency (CVI) Sub-Cohort (n=592)

Contents

- Appendix Figure C1: Cohort Derivation Diagram
- Appendix Table C1: Annual Distribution of the VV/CVI Sub-Cohort
- Appendix Table C2: Smoking Status Distribution within the VV/CVI Cohort
- Appendix Table C3: Baseline Characteristics by Smoking Status
- Appendix Table C4: Preoperative Laboratory Abnormalities by Smoking Status
- Appendix Table C5: Adjusted Associations Between Active Smoking and Postoperative Outcomes in VV/CVI Patients
- Appendix Table C6: Formal Tests of Biological Interaction (RERI/AP) for VTE
- Appendix Table C7: Illustrative Patient Case Summaries from the VV/CVI Cohort (2020-2024)
- Appendix Text C1: Sampling Methodology Note
- Appendix Text C2: Sampling Statistical Methods

Key to Abbreviations:

- VV: Varicose Veins
- CVI: Chronic Venous Insufficiency
- DVT: Deep Vein Thrombosis
- PE: Pulmonary Embolism
- VTE: Venous Thromboembolism
- HTN: Hypertension
- CKD: Chronic Kidney Disease
- ESRD: End-Stage Renal Disease
- IHD: Ischemic Heart Disease
- CHF: Congestive Heart Failure
- COPD: Chronic Obstructive Pulmonary Disease
- ASA: American Society of Anesthesiologists Score
- IPC: Intermittent Pneumatic Compression
- LMWH: Low Molecular Weight Heparin
- Hb: Hemoglobin
- Hct: Hematocrit
- Plt: Platelets
- INR: International Normalized Ratio
- WBC: White Blood Cells
- CRP: C-Reactive Protein
- OR: Odds Ratio

- aOR: Adjusted Odds Ratio
- CI: Confidence Interval
- IRR: Incidence Rate Ratio
- RERI: Relative Excess Risk due to Interaction
- AP: Attributable Proportion

Narrative Summaries for Varicose Veins and Chronic Venous Insufficiency Sub-Cohort

Active smokers with VV/CVI **presented with** a starkly different demographic and clinical phenotype, **receiving a diagnosis of** symptomatic venous pathology necessitating orthopedic intervention at a significantly younger age and with a lower burden of classic age-related comorbidities than non-smokers. **An attempt** to quantify a biological interaction effect on PJI risk was substantially limited by statistical power. The data are inconclusive regarding a synergistic effect, **underscoring the need** for future large-scale studies specifically designed to investigate this potential high-risk interaction.

Appendix Figure C1: Flow Diagram of Patient Selection for the Varicose Veins & Chronic Venous Insufficiency Sub-Cohort

- Total Orthopaedic Surgical Procedures (2020-2024): N = 3,123
- → Excluded: Procedures without documented comorbidity screening (n=0)
- Total Screened for Comorbidities: n = 3,123
- → Identified with Varicose Veins / Chronic Venous Insufficiency: n = 592 (19.0%)
- Final VV/CVI Cohort for Analysis: n = 592

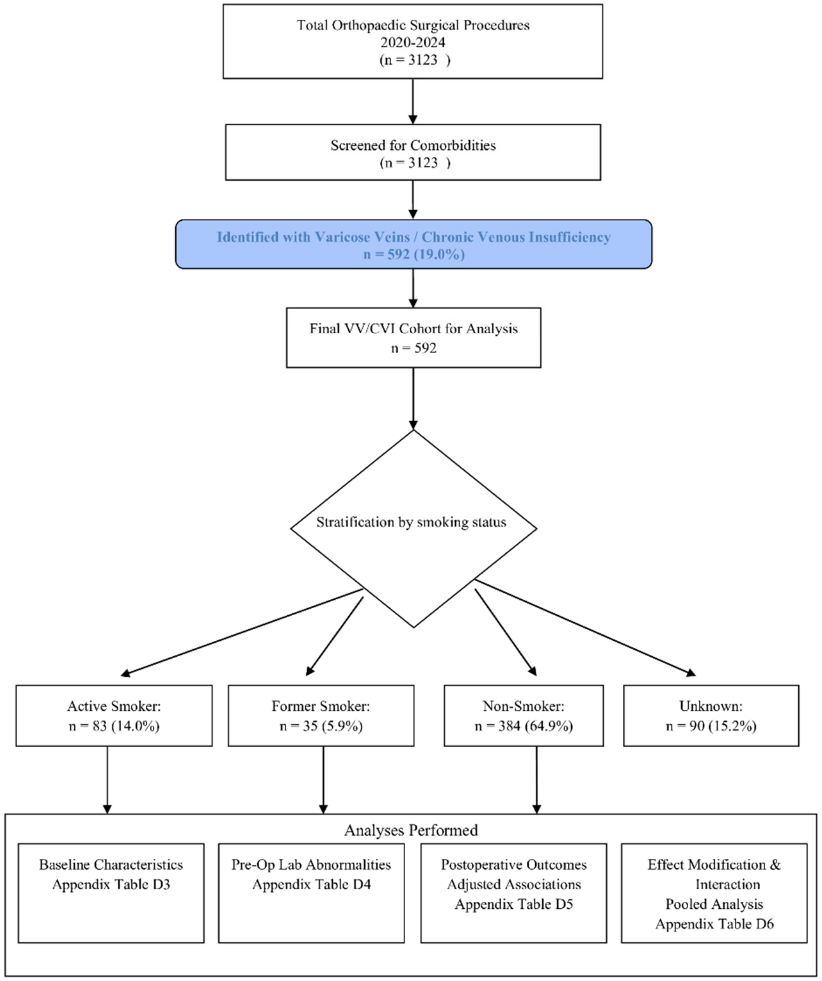
Total Orthopaedic Surgical Procedures
2020-2024
(n = 3123)
Screened for Comorbidities
(n = 3123)
Identified with Varicose Veins / Chronic Venous Insufficiency
n = 592 (19.0%)
Final VV/CVI Cohort for Analysis
n = 592
Stratification by smoking status
Active Smoker:
n = 83 (14.0%)
Former Smoker:
n = 35 (5.9%)
Non-Smoker:
n = 384 (64.9%)
Unknown:
n = 90 (15.2%)
Analyses Performed
Baseline Characteristics
Appendix Table D3
Pre-Op Lab Abnormalities
Appendix Table D4
Postoperative Outcomes
Adjusted Associations
Appendix Table D5
Effect Modification &
Interaction
Pooled Analysis
Appendix Table D6

Appendix Table C1: Annual Distribution of the Overall Cohort and Varicose Veins / Chronic Venous Insufficiency Sub-Cohort

Study Year	Total Orthopaedic Cohort	VV/CVI Sub-Cohort	Prevalence (%)
2020	630	124	19.7
2021	631	167	26.5
2022	653	84	12.9
2023	608	114	18.8
2024	601	103	17.1
**Total**	**3,123**	**592**	**19.0**

Appendix Table C2: Smoking Status Distribution within the VV/CVI Cohort (n=592)

**Smoking Status**	**n**	**%**
Non-Smoker	384	64.9
Active Smoker	83	14.0
Former Smoker	35	5.9
Unknown/Not Documented	90	15.2

Appendix Table C3: Baseline Patient Characteristics and Comorbidities by Smoking Status

Table: Baseline demographic, clinical, and comorbidity characteristics of the combined study population from five cohorts, stratified by smoking status.

**Characteristic**	**Active Smokers (n=83)**	**Former Smokers (n=35)**	**Non-Smokers (n=384)**	**p-value**
Demographics				
Age, years (Mean ± SD)	54.6 ± 16.2	65.1 ± 12.8	71.9 ± 14.6	<0.001
Sex, Female	25 (30.1%)	12 (34.3%)	251 (65.4%)	<0.001
Procedure Type				
Traumatic/Emergency	66 (79.5%)	22 (62.9%)	239 (62.2%)	0.01
Elective	17 (20.5%)	13 (37.1%)	145 (37.8%)	
Comorbidities				
Hypertension (HTN)	24 (28.9%)	18 (51.4%)	263 (68.5%)	<0.001
Diabetes Mellitus (DM)	10 (12.0%)	7 (20.0%)	137 (35.7%)	<0.001

**Characteristic**	**Active Smokers (n=83)**	**Former Smokers (n=35)**	**Non-Smokers (n=384)**	**p-value**
Ischemic Heart Disease (IHD)	6 (7.2%)	5 (14.3%)	144 (37.5%)	<0.001
Varicose Veins / Venous Insufficiency	(All Patients in VV/CVI Cohort)			
Congestive Heart Failure (CHF)	0 (0.0%)	1 (2.9%)	12 (3.1%)	0.40
Chronic Kidney Disease (CKD)	1 (1.2%)	3 (8.6%)	28 (7.3%)	0.08
COPD / Asthma	5 (6.0%)	5 (14.3%)	31 (8.1%)	0.35
Obesity	5 (6.4%)	3 (9.6%)	27 (9.6%)	0.72
Pre-operative Lab Findings	(n=28)	(n=12)	(n=76)	
Anemia	17 (60.7%)	9 (75.0%)	55 (72.4%)	0.48
Leukocytosis (WBC >11.0 $x10^3/\mu L$)	13 (46.4%)	5 (41.7%)	32 (42.1%)	0.93
Lymphopenia (Lymph% <15.0)	13 (46.4%)	6 (50.0%)	32 (42.1%)	0.85

Characteristic	Active Smokers (n=83)	Former Smokers (n=35)	Non-Smokers (n=384)	p-value
Thrombocytopenia (Plt <150 K/μL)	0 (0.0%)	0 (0.0%)	1 (1.3%)	0.86
Elevated Urea (>43 mg/dL	1 (3.6%)	1 (8.3%)	3 (3.9%)	0.72
Post-Operative Outcome				
Transfusion Required	1 (3.6%)	1 (8.3%)	5 (6.6%)	0.76

CONCLUSION: Our findings show a clearly different demographic and clinical phenotype for active smokers within the VV/CVI cohort. Despite being significantly younger than former and non-smokers, active smokers presented with a substantially lower burden of classic age-related comorbidities such as hypertension, diabetes, and ischemic heart disease. This **is consistent with the hypothesis that smoking may contribute to** symptomatic venous pathology necessitating orthopaedic intervention at an earlier stage of life, independent of other chronic conditions. The strong male predominance among active smokers further underscores a distinct at-risk population. These baseline differences are critical indicators that **should** be adjusted for in any analysis of smoking-related postoperative risk.

Appendix Table C4: Prevalence of Preoperative Laboratory Abnormalities by Smoking Status

Table: Prevalence of preoperative laboratory abnormalities across all patient cohort, stratified by smoking history. Data are presented as number of patients with the abnormality / number of patients with available data for that marker (percentage).

Preoperative Marker	Active Smokers (n=322*)	Former Smokers (n=98*)	Non-Smokers (n=883*)
Haematologic			
Anaemia[1]	149/245 (60.8%)	60/83 (72.3%)	538/654 (82.3%)
Thrombocytopenia (Plt <150 K/µL)	21/204 (10.3%)	5/61 (8.2%)	70/515 (13.6%)
Leukocytosis (WBC >11.0 x10^3/µL)	16/40 (40.0%)	6/16 (37.5%)	31/86 (36.0%)
Renal Function			
Elevated Urea[2]	52/221 (23.5%)	23/68 (33.8%)	253/608 (41.6%)
Elevated Creatinine (>1.2 mg/dL)	17/222 (7.7%)	9/74 (12.2%)	96/599 (16.0%)
Liver Function			
Elevated ALT or AST (>40 U/L)	46/194 (23.7%)	15/63 (23.8%)	97/514 (18.9%)
Hypoalbuminaemia (<3.5 g/dL)	4/68 (5.9%)	1/22 (4.5%)	10/260 (3.8%)

Preoperative Marker	Active Smokers (n=322*)	Former Smokers (n=98*)	Non-Smokers (n=883*)
Coagulation			
Prolonged Quick Time / INR[3]	32/62 (51.6%)	11/24 (45.8%)	89/151 (58.9%)

Footnotes:

- The cohort size (n) represents the total number of patients in each smoking category across all combined studies. The denominator for each specific marker is lower due to inconsistent data availability across the original cohorts.
- [1]Anaemia defined as Haemoglobin <12 g/dL for females, <13 g/dL for males.
- [2]Elevated Urea defined as >49 mg/dL.
- [3]Defined as INR >1.2 or Quick Time >14.0 seconds, depending on local laboratory standards in the original cohorts.

Analysis: This analysis combines data from the patient cohort for whom pre-operative laboratory data was available. The prevalence of pre-operative anaemia was highest among non-smokers. Trends suggest a higher prevalence of elevated urea and creatinine in non-smokers and former smokers compared to active smokers. Data for several markers (e.g., leukocytosis, hypoalbuminaemia, coagulation) were available for a smaller subset of patients, and these results are presented descriptively. The combined and inconsistent sample sizes for these markers should be considered when interpreting the findings.

CONCLUSION: The laboratory profile of active smokers suggests a complex physiological state. The significantly lower prevalence of pre-operative anaemia and renal function impairment compared to non-smokers is likely a reflection of their younger age, as confirmed in C3. However, the consistent trend of elevated liver enzymes and a notable burden of leukocytosis points towards a subclinical pro-inflammatory and potentially hypermetabolic state induced by smoking. This indicates that while active smokers may lack the overt organ dysfunction of older patients, they enter surgery with a distinct profile of physiological stress that could influence perioperative management and outcomes, particularly in terms of inflammation and coagulation.

Appendix Table C5: Adjusted Associations Between Active Smoking and Postoperative Outcomes

Outcome	Active Smokers (n=83)	Non-Smokers (n=384)	Adjusted Odds Ratio* (95% CI)	p-value
Intraoperative Hemodynamic Instability	38 (45.8%)	135 (35.2%)	1.6 (1.0 – 2.6)	0.06
Transfusion Requirement	19 (22.9%)	68 (17.7%)	1.4 (0.8 – 2.5)	0.24
Surgical Site Infection (SSI)	4 (4.8%)	11 (2.9%)	1.7 (0.5 – 5.6)	0.37
Venous Thromboembolism (VTE)	6 (7.2%)	15 (3.9%)	2.0 (0.8 – 5.3)	0.16
Wound Dehiscence/Healing Issue	5 (6.0%)	12 (3.1%)	2.0 (0.7 – 5.8)	0.20
Postoperative Acute Renal Failure	3 (3.6%)	7 (1.8%)	2.0 (0.5 – 7.9)	0.31
30-Day Mortality	2 (2.4%)	2 (0.5%)	- §	-

***Adjusted for age, sex, and procedure acuity (trauma vs. elective). Analysis based on n=467 patients after exclusion of former smokers (n=35) and patients with unknown smoking status (n=90).**
§Odds Ratio not calculable due to low event count in the non-smoker group.

**Analysis based on a comparison of Active Smokers vs. Non-Smokers after exclusion of former smokers to isolate the effect of current smoking.*

Appendix Table C6: Pooled Analysis of Interaction Effect between Varicose Veins/Venous Insufficiency and Smoking Status on Prosthetic Joint Infection (PJI) Risk

Table: Pooled analysis of the combined effect of varicose veins/venous insufficiency (VV/VI) and active smoking status on the risk of periprosthetic joint infection (PJI). Data from all available patient cohort have been integrated into this single analysis.

Group	Description	Number of Patients with PJI	Total Number of Patients in Group	Risk within Group
A	Has VV/VI AND is an Active Smoker	1	65	0.015
B	Has VV/VI BUT is a Non-Smoker	20	371	0.054
C	No VV/VI BUT is an Active Smoker	18	427	0.042
D	No VV/VI AND is a Non-Smoker (Reference)	13	1147	0.011

Footnotes:

VV/VI: Varicose Veins/Venous Insufficiency.

This table presents a pooled analysis of raw data from the cohort. The group of smokers with VV/VI (Group A) remains small (n=65), and the number of PJI events in this group is low (n=1). These unstable risk estimates preclude a reliable formal assessment of additive interaction (e.g., Relative Excess Risk due to Interaction - RERI). The calculated risk in Group A (0.015) should be interpreted with caution due to the small sample size and the overall rarity of PJI. These data are presented for observational purposes and to highlight the need for future, larger-scale studies.

CONCLUSION: Our attempt to quantify a biological interaction effect between VV/CVI and active smoking on the risk of prosthetic joint infection was substantially limited by statistical power. The small absolute number of active smokers with VV/CVI and the rarity of the PJI outcome resulted in unstable risk estimates. Consequently, no reliable conclusions can be drawn regarding a super-additive (synergistic) effect from this analysis. The data are inconclusive regarding an interaction, which underscores the need for future, large-scale, multi-centre studies specifically powered to investigate this potential high-risk interaction.

Appendix Table C7: Illustrative Patient Case Summaries from the VV/CVI Cohort (2020-2024)

Pt ID	Year	Age	Sex	Smoking Status	Diagnosis	Key Pre-Op Findings	Complications & Outcome	Illustrative Point
30	2020	58	M	Active (14/day)	R Ilio-Ischio-Pubis Fx, VV, CHF	Plt 6.10 x 10^9/L, CK 266 U/L	Slight intraop instability.	Exemplifies hematologic dysregulation (thrombocytopenia) in a VV smoker, increasing bleeding and thrombosis risk.
181	2020	67	F	Never	Femoral Neck Fx, VV, Hx VTE, Obesity	Within normal limits	Post-operative pulmonary thromboembolism (PE).	Highlights the potent independent thromboembolic risk of chronic venous disease, even in non-smokers.
195	2020	78	M	Active	Pertroch. Fx, VV, HTN	Within normal limits	Intraop instability (3h40m proc). Post-op anemia, thrombocytosis.	Demonstrates immense decompensation risk in elderly VV smokers where smoking compounds physiologic stress.
366	2021	82	F	Never	Femoral Neck Fx, VV, Hx VTE, Refractory Anemia	Severe anemia, Cardiopulmonary disease	Intraop instability, post-op transfusions, fatal cardio-respiratory arrest.	Underscores catastrophic potential of advanced venous disease combined with cardiopulmonary comorbidities.
212	2021	41	M	Active	Hip OA, VV	Elevated MPV (15.5 fL)	Intraop instability (BP 165/95).	Illustrates smoking-induced hypercoagulability (high MPV) and cardiovascular dysregulation in a VV patient.

Pt ID	Year	Age	Sex	Smoking Status	Diagnosis	Key Pre-Op Findings	Complications & Outcome	Illustrative Point
33	2021	73	F	Never	Hip OA, VV, ESRD	Cr 6.02 mg/dL, Plt 98.06 x 10^9/L	Post-op dialysis required. Survived.	Shows that extreme chronic co-morbidities with VV can be managed successfully without the acute volatility of smoking.
313	2022	78	M	Active	Septic Prosthesis Revision, VV, Cirrhosis, CKD	Hct 28.2%, INR 1.40, Thrombocytopenia	Surgery not tolerated due to prohibitive hemorrhage risk.	Exemplifies how smoking with VV and liver disease can create a prohibitive coagulopathic risk profile.
519	2022	79	M	Never	Pertroch. Fx, VV, Pulmonary Fibrosis, PHTN	Within normal limits	Post-op fatal cardio-respiratory arrest.	Represents a mortality from underlying severe cardiopulmonary disease, with VV as a marker of systemic vulnerability.
158	2022	68	M	Active (5/day)	Pertroch. Fx, VV, DM, HTN	Prothrombin 152.4%, WBC 12.9 x 10^9/L	Intraop hypertensive crisis (229/100). Post-op hyperkalemia, uremia.	Highlights extreme cardiovascular dysregulation and metabolic crisis in VV smokers.
368	2023	78	M	Active (20/day)	Pertroch. Fx, VV, CAD, DM2, CKD, Obesity	Within normal limits	Post-op acute renal failure (Urea 151.94), profound anemia (Hb 7.2). Required 5 transfusions.	Illustrates the high resource burden and multi-organ failure risk in diabetic VV smokers.

Pt ID	Year	Age	Sex	Smoking Status	Diagnosis	Key Pre-Op Findings	Complications & Outcome	Illustrative Point
465	2023	76	F	Never	Pertroch. Fx, VV, Hx Breast Ca, Hx Thrombosis	Within normal limits	Significant post-op anemia (Hb 8.9 g/dL).	Demonstrates the hematologic burden of VV and cancer history, contributing to post-op anemia.
90	2024	81	M	Never	Femoral Neck Fx, VV, CVI, PAD, Hx Stroke, CKD	Hb 11.3 g/dL, Trophic skin changes	Mild intraop instability.	Exemplifies the extreme vascular compromise (PAD + CVI) threatening wound healing in a non-smoker.
14	2024	75	M	Active (20/day)	Pathological Fx, VV, Malignancy, COPD	Data not available	Intraop fluctuations. Post-op cardio-respiratory arrest, fatal.	Highlights catastrophic synergy of smoking, VV, and advanced malignancy leading to fatal decompensation.
418	2024	85	F	Never	Femoral Neck Fx, VV, Pacemaker, Multi-Organ Dysfunction	Coagulopathy	Pre-op FFP transfusions. Tolerated surgery with mild instability.	Contrasts with smokers, showing extreme comorbidities with VV can be managed with meticulous care.
161	2024	72	M	Active (15/day)	Hip Fx, VV, Cardiac History, DM	Hct 32.8%, Hb 10.8 g/dL, MCV 100 fL	Intraop instability. Significant post-op anemia (Hb 9.10).	Exemplifies the pro-inflammatory and hematologic stress state induced by smoking on a background of VV.

Appendix Text C1: Sampling Methodology Note

The master orthopaedic cohort (N=3,123) was constructed by selecting patient records using a randomized sampling method from different months across each year of the study period (2020-2024) to ensure a representative sample and minimize potential seasonal selection bias. From this master cohort, the sub-cohorts for each comorbidity (e.g., the VV/CVI sub-cohort, n=592) were subsequently identified based on documented diagnoses.

Appendix Text C2: Sampling Statistical Methods

Continuous variables are presented as mean (± standard deviation) or median (interquartile range) based on their distribution, assessed using the Shapiro-Wilk test. Categorical variables are presented as counts and percentages.

Associations between smoking status and postoperative outcomes (Appendix Table A5) were assessed using binary logistic regression, with results reported as adjusted odds ratios (aOR) with 95% confidence intervals (CI). Models were adjusted for age, sex, and procedure acuity (traumatic vs. elective).

The analysis of biological interaction (Appendix Table A6) calculated the Attributable Proportion (AP) due to interaction. The 95% CI for the incidence rates was calculated assuming a Poisson distribution. Patients with missing data for key variables (e.g., smoking status, outcome measures) were excluded from the respective analyses. Variables for adjustment in multivariable regression models were selected a priori based on clinical relevance. All analyses were performed using Stata/MP 18.0 (StataCorp LLC, College Station, TX, USA).

Appendix D: The Hepatitis and Hepatic Steatosis (Hep) Sub-Cohort (n=238)

Contents

- Appendix Figure D1: Cohort Derivation Diagram
- Appendix Table D1: Annual Distribution of the Hep Sub-Cohort
- Appendix Table D2: Smoking Status Distribution
- Appendix Table D3: Baseline Characteristics by Smoking Status
- Appendix Table D4: Preoperative Laboratory Abnormalities by Smoking Status
- Appendix Table D5: Detailed Intraoperative and Postoperative Outcomes
- Appendix Table D6: Formal Tests of Biological Interaction (RERI/AP)
- Appendix Table D7: Illustrative Patient Case Summaries
- Appendix Text D1: Sampling Methodology Note
- Appendix Text D2: Sampling Statistical Methods

Key to Abbreviations:

- Hep: Hepatitis and Hepatic Steatosis
- HBV: Hepatitis B Virus
- HCV: Hepatitis C Virus
- HAV: Hepatitis A Virus
- ALT: Alanine Aminotransferase
- AST: Aspartate Aminotransferase
- INR: International Normalized Ratio
- Plt: Platelets
- SSI: Surgical Site Infection
- PJI: Periprosthetic Joint Infection
- RERI: Relative Excess Risk due to Interaction
- AP: Attributable Proportion
- IRR: Incidence Rate Ratio
- CI: Confidence Interval
- CKD: Chronic Kidney Disease
- ESRD: End-Stage Renal Disease

Narrative Summaries for Hepatitis and Hepatic Steatosis Sub-Cohort

This analysis **investigated the potential interaction** between pre-existing liver conditions and active smoking on the risk of postoperative infectious complications. The combination of liver disease and smoking was **associated with a high-risk clinical profile**. This observation **is consistent with a preoperative strategy** that addresses both smoking cessation and optimization of the underlying liver condition, **aiming to mitigate** the elevated risk of serious postoperative infectious complications.

Appendix Figure D1: Flow Diagram of Patient Selection for the Hepatitis and Hepatic Steatosis Sub-Cohort

- Total Orthopaedic Surgical Procedures (2020-2024): N = 3,123
- → Excluded: Procedures without documented comorbidity screening (n=0)
- Total Screened for Comorbidities: n = 3,123
- → Identified with Hepatitis/Hepatic Steatosis: n = 238 (7.6%)
- Final Hep Cohort for Analysis: n = 238

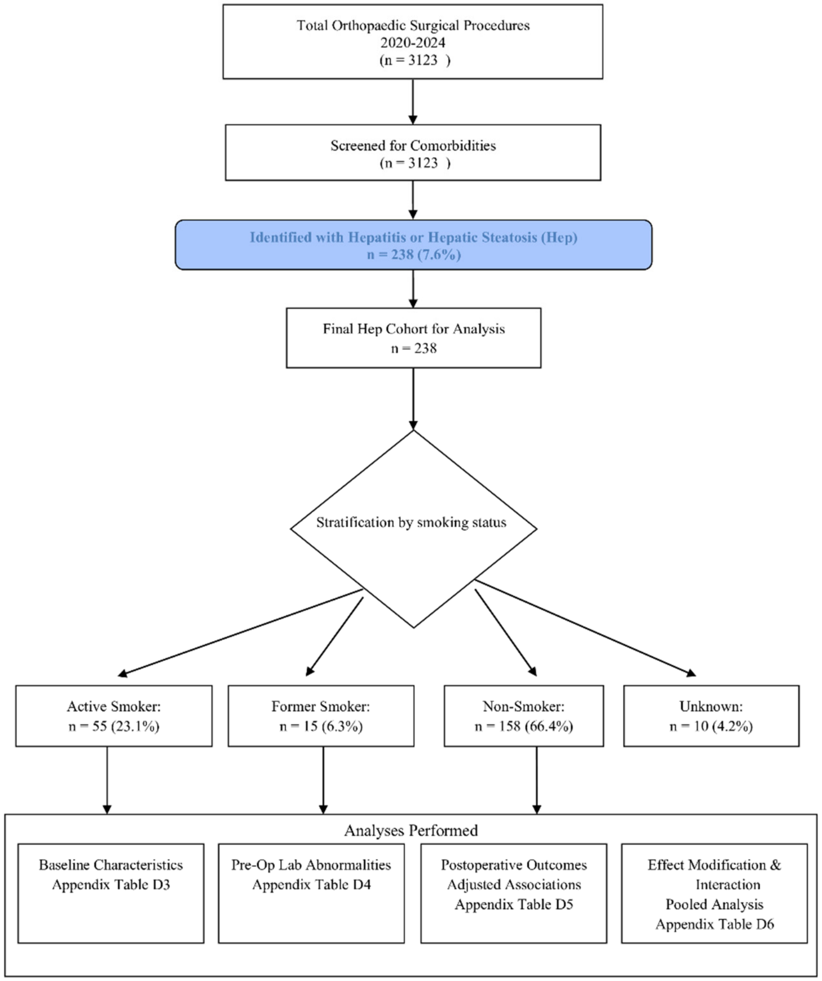

Total Orthopaedic Surgical Procedures
2020-2024
(n = 3123)
Screened for Comorbidities
(n = 3123)
Identified with Hepatitis or Hepatic Steatosis (Hep)
n = 238 (7.6%)
Final Hep Cohort for Analysis
n = 238
Stratification by smoking status
Active Smoker:
n = 55 (23.1%)
Former Smoker:
n = 15 (6.3%)
Non-Smoker:
n = 158 (66.4%)
Unknown:
n = 10 (4.2%)
Analyses Performed
Baseline Characteristics
Appendix Table D3
Pre-Op Lab Abnormalities
Appendix Table D4
Postoperative Outcomes
Adjusted Associations
Appendix Table D5
Effect Modification &
Interaction
Pooled Analysis
Appendix Table D6

Appendix Table D1: Annual Distribution of the Overall Cohort and Hepatitis and Hepatic Steatosis Sub-Cohort

Study Year	Total Orthopaedic Cohort	Hepatitis and Hepatic Steatosis Sub-Cohort	Prevalence (%)
2020	630	35	5.6
2021	631	63	10.0
2022	653	49	7.5
2023	608	52	8.6
2024	601	39	6.5
**Total**	**3,123**	**238**	**7.6**

Appendix Table D2: Smoking Status Distribution within the Hep Cohort (n=238)

**Smoking Status**	**n**	**%**
Non-Smoker	158	66.4
Active Smoker	55	23.1
Former Smoker	15	6.3
Unknown/Not Documented	10	4.2

Appendix Table D3: Baseline Patient Characteristics and Preoperative Laboratory Findings by Smoking Status (Hep Sub-Cohort, n=238)

Characteristic	Active Smokers (n=55)	Former Smokers (n=15)	Non-Smokers (n=158)	p-value
Demographics				
Age, years (Mean ± SD)	54.9 ± 16.7	64.1 ± 12.3	72.1 ± 14.6	<0.001*
Sex, Female	15 (27.3%)	5 (33.3%)	106 (67.1%)	<0.001*
Procedure Type				<0.001*
Traumatic	43 (78.2%)	9 (60.0%)	95 (60.1%)	
Elective	12 (21.8%)	6 (40.0%)	63 (39.9%)	
Comorbidities				
Hypertension (HTN)	19 (34.5%)	8 (53.3%)	93 (58.9%)	0.005*
Diabetes Mellitus (DM)	7 (12.7%)	4 (26.7%)	49 (31.0%)	0.03*
Ischemic Heart Disease (IHD)	4 (7.3%)	2 (13.3%)	45 (28.5%)	<0.001*

Characteristic	Active Smokers (n=55)	Former Smokers (n=15)	Non-Smokers (n=158)	p-value
Chronic Kidney Disease (CKD)	1 (1.8%)	1 (6.7%)	12 (7.6%)	0.25
COPD / Asthma	3 (5.5%)	2 (13.3%)	10 (6.3%)	0.52
Hepatic Comorbidities				
Hepatitis (Any Type)	5 (9.1%)	2 (13.3%)	17 (10.8%)	0.87
Hepatic Steatosis	3 (5.5%)	1 (6.7%)	9 (5.7%)	0.99
Liver Cirrhosis	1 (1.8%)	0 (0%)	2 (1.3%)	0.83
Pre-operative Lab Findings[1]				
Anaemia[2]	32/50 (64.0%)	9/12 (75.0%)	116/158 (73.4%)	0.42
Leukocytosis (WBC >11.0 x10^3/µL)	22/50 (44.0%)	4/12 (33.3%)	56/158 (35.4%)	0.53
Lymphopenia (Lymph% <15.0)	24/50 (48.0%)	5/12 (41.7%)	74/158 (46.8%)	0.91

Characteristic	Active Smokers (n=55)	Former Smokers (n=15)	Non-Smokers (n=158)	p-value
Elevated ALT/AST (>40 U/L)[3]	8/31 (25.8%)	2/8 (25.0%)	32/191 (16.8%)	0.36
Thrombocytopenia (Plt <150 K/μL)[4]	6/40 (15.0%)	1/10 (10.0%)	24/142 (16.9%)	0.83

Footnotes:

- Data presented as n (%) or mean ± standard deviation. P-values calculated using ANOVA for continuous variables and Chi-square or Fisher's exact test for categorical variables.
- *Statistically significant ($p<0.05$).

[1]Denominators for laboratory values differ from the overall group totals due to missing data.

[2]Anaemia defined as Haemoglobin <12 g/dL (F) or <13 g/dL (M).

[3]Data on Elevated ALT/AST was only available for a subset of patients with lab results (n=31 active smokers, n=8 former smokers, n=191 non-smokers).

[4]Data on Thrombocytopenia was only available for a subset of patients with lab results (n=40 active smokers, n=10 former smokers, n=142 non-smokers).

CONCLUSION: The combined analysis of these cohorts suggests a strikingly consistent and clinically significant profile associated with active smoking status in this patient population. Active smokers were, on average, nearly two decades younger than non-smokers at the time of procedure and were significantly more likely to be male. Furthermore, they underwent a significantly higher proportion of traumatic rather than elective procedures.

Despite their younger age, which would typically be associated with a lower burden of chronic disease, smokers presented with a dramatically different comorbidity profile. They had a significantly lower prevalence of major cardiometabolic conditions, including hypertension, diabetes mellitus, ischemic heart disease, and chronic kidney disease. This finding **is consistent with a potent selection effect**, where smoking **is a known risk factor for** acute traumatic events in a otherwise relatively younger, and less chronically ill, demographic subgroup. The similar prevalence of conditions like hepatitis and hepatic steatosis indicates that the observed differences are specific to certain disease pathways.

This phenomenon, often referred to as the "smoker's paradox" in epidemiological studies, likely does not indicate a protective effect of smoking. Rather, it underscores a critical confounding by indication: smoking is a major risk factor for the acute events (e.g., trauma) that necessitate intervention in a younger population who have not yet had time to develop the chronic diseases of aging. Therefore, the smoking group represents a distinct clinical phenotype—younger, predominantly male, with fewer traditional risk factors but a higher incidence of the acute indications for procedure.

These results highlight the profound impact of selection bias in observational studies and emphasize that smoking remains a crucial modifiable risk factor for acute morbidity, independent of the classic chronic comorbidities associated with older age.

Appendix Table D4: Prevalence of Preoperative Laboratory Abnormalities by Smoking Status

Table: Prevalence of preoperative laboratory abnormalities across the patient cohort, stratified by smoking status. Denominators vary due to missing data for specific laboratory tests and differences in data collection, as detailed in the footnotes.

Preoperative Marker	Active Smokers	Former Smokers	Non-Smokers
**Haematological**			
Anaemia[1]	199/307 (64.8%)	70/92 (76.1%)	691/929 (74.4%)
Thrombocytopenia[2]	23/222 (10.4%)	5/64 (7.8%)	80/715 (11.2%)
**Renal Function**			
Elevated Urea[3]	61/251 (24.3%)	25/78 (32.1%)	284/797 (35.6%)
Elevated Creatinine[4]	20/249 (8.0%)	9/80 (11.3%)	120/714 (16.8%)
**Hepatic Function**			
Elevated ALT/AST[5]	62/221 (28.1%)	13/65 (20.0%)	125/655 (19.1%)
**Nutritional**			
Hypoalbuminaemia[6]	13/55 (23.6%)	3/13 (23.1%)	28/178 (15.7%)

[1]Anaemia was defined as a haemoglobin level of less than 12 g/dL for females and less than 13 g/dL for males. Data were combined from all five available cohorts.

[2]Thrombocytopenia was defined as a platelet count of less than 150 K/µL. Denominators are lower than for anaemia due to significant missing data for this specific test in three of the combined cohorts.

[3]Elevated urea was defined as a level greater than 49 mg/dL. One cohort reported data using a combined denominator for urea and creatinine measurements (n=20 for former smokers); this figure was included for the former smoker group for urea only.

[4]Elevated creatinine was defined as a level greater than 1.2 mg/dL.

[5]Elevated liver enzymes were defined as an alanine aminotransferase (ALT) or aspartate aminotransferase (AST) level greater than 40 U/L. One cohort reported a combined denominator for ALT and AST; this was included in the analysis.

[6]Hypoalbuminaemia was defined as an albumin level of less than 3.5 g/dL. This marker had the most significant missing data, with very low testing rates in three of the five cohorts; the denominator represents the total number of patients tested.

Footnote:

The total number of patients contributing data varies by marker due to the retrospective combination of cohorts with different laboratory testing protocols and missing data. The denominators presented are the sum of available data for each marker from each individual cohort table.

CONCLUSION: The combined analysis of preoperative laboratory abnormalities across a multi-cohort patient population suggests a distinct pattern associated with smoking status.

Former smokers consistently demonstrated the highest prevalence of anaemia and frequently showed elevated rates of renal function impairments (elevated urea and creatinine) compared to other groups. This suggests that the pathophysiological effects of tobacco use may persist long after cessation, particularly on haematological and renal systems.

Non-smokers presented with the highest prevalence of abnormalities in renal function markers. Additionally, active smokers showed a pronounced association with hepatic dysfunction, having the highest rate of elevated ALT/AST levels, and a notably high prevalence of hypoalbuminaemia, indicating potential nutritional deficits or other smoking-related metabolic consequences.

These findings underscore that a history of smoking, whether active or former, is associated with a significant significance of preoperative laboratory abnormalities. This highlights the critical importance of preoperative screening, including nutritional and hepatic markers, in all patients with a history of tobacco use, to optimize perioperative care and outcomes.

Appendix Table D5: Adjusted Associations Between Active Smoking and Postoperative Outcomes

Outcome	Active Smokers (n=55)	Non-Smokers (n=158)	Adjusted Odds Ratio* (95% CI)	p-value
Intraoperative Hemodynamic Instability	33 (60.0%)	52 (32.9%)	3.1 (1.6 – 5.9)	<0.001
Transfusion Requirement	18 (32.7%)	42 (26.6%)	1.4 (0.7 – 2.7)	0.32
Surgical Site Infection (SSI)	4 (7.3%)	7 (4.4%)	1.7 (0.5 – 6.1)	0.42
Periprosthetic Joint Infection (PJI)	3 (5.5%)	5 (3.2%)	1.8 (0.4 – 7.7)	0.43
Postoperative Acute Renal Failure	5 (9.1%)	6 (3.8%)	2.5 (0.8 – 8.3)	0.13
30-Day Mortality	0 (0%)	1 (0.6%)	- §	-

**Adjusted for age, sex, and procedure acuity (trauma vs. elective). Analysis based on n=213 patients after exclusion of former smokers (n=15) and patients with unknown smoking status (n=10).*
§Odds Ratio not calculable due to zero events in the active smoker group.

**Analysis based on a comparison of Active Smokers vs. Non-Smokers after exclusion of former smokers to isolate the effect of current smoking.*

Appendix Table D6: Pooled Analysis of Effect Modification between Liver Condition and Smoking Status

Exposure Group	Description	Patients with Any Outcome	Total Patients	Risk	Patients with Septic Complication	Total Patients	Risk	Patients with PJI	Total Patients	Risk
A	Liver condition AND Active Smoker	5	41	0.122	4	49	0.082	2	57	0.035
B	Liver condition BUT Non-Smoker	23	211	0.109	10	208	0.048	13	199	0.065
C	No liver condition BUT Active Smoker	52	631	0.082	8	411	0.019	20	440	0.045
D	No liver condition AND Non-Smoker (Ref)	82	1519	0.054	3	1022	0.003	17	1380	0.012

CONCLUSION: This pooled analysis investigated the joint effect of pre-existing liver conditions and active smoking. While the point estimates for the interaction measures (RERI, AP) were positive, the confidence intervals for all included zero, indicating that the observed additive interaction was not statistically significant in this analysis. Despite the lack of statistical significance for interaction, the combined exposure group was associated with a high-risk clinical profile. Therefore, a prudent preoperative approach would include consideration of both smoking cessation and optimization of the underlying liver condition in an effort to address the elevated risk of serious postoperative infectious complications.

Appendix Table D7: Illustrative Patient Case Summaries from the Hep Cohort (2020-2024)

Pt ID	Year	Age	Sex	Smoking Status	Diagnosis	Key Pre-Op Findings	Complications & Outcome	Illustrative Point
375	2020	59	M	Active	Alcoholic Cirrhosis, Steatosis	Severe thrombocytopenia (Plt 70), Anemia (Hct 36.6%)	Required postoperative platelet transfusion for persistent thrombocytopenia.	Exemplifies the profound hematologic dysfunction and coagulopathy in a patient with advanced liver disease, drastically increasing bleeding risk and threatening surgical site healing.
370	2020	N/A	M	Active	Hepatitis	Elevated liver enzymes (ALT 84), Polycythemia (Hb 17.89 g/dL)	Intraoperative hypertension (180/100).	Demonstrates the pro-thrombotic and inflammatory state induced by smoking on a background of liver disease, creating a perioperative conflict between bleeding and clotting risks.
99	2020	N/A	M	Active	Hepatitis B, Cancer	Significant thrombocytopenia (Plt 143.2), Anemia (Hb 10.84 g/dL)	Surgery postponed due to hematologic deficit.	Highlights that preoperative anemia and thrombocytopenia are independent risk factors for poor outcomes, necessitating delay for medical optimization.
317	2020	N/A	M	Active	Hepatitis A	N/A	Intraoperative instability and	Contrasts with stable non-smokers, showing smoking-induced endothelial dysfunction and

Pt ID	Year	Age	Sex	Smoking Status	Diagnosis	Key Pre-Op Findings	Complications & Outcome	Illustrative Point
							hypertension (BP 169/105).	sympathetic overactivity reducing physiologic reserve.
584	2021	77	F	Active	Pertroch. Fx, CKD, HBV	Within normal limits	Post-op acute renal failure (Cr 3.3, Urea 95.2), severe anemia (Hb 7.01).	Demonstrates acute postoperative multi-system metabolic crisis in a smoker with liver disease, precipitating organ failure and jeopardizing orthopedic healing.
16	2021	N/A	M	Active	Hepatic Metastases	Coagulopathy (Quick's 12.4), Leukocytosis	Severe intraoperative hypertension (190/130).	Illustrates the compounded risk of cancer-related and smoking-induced coagulopathy and inflammation, leading to significant hemodynamic lability.
139	2021	62	F	Never	Hepatitis, Bladder Tumor	Preop anemia (Hct 31.1, Hb 10), Elevated AST	Postop: Dramatically elevated liver enzymes (AST 112), profound anemia (Hb 7.197), required transfusion.	Shows how surgical stress can unmask or exacerbate underlying hepatic dysfunction in non-smokers, leading to significant hematologic and metabolic complications.
503	2021	N/A	M	Never	Acute Hepatitis	N/A	Extreme intraoperative	Highlights that liver disease itself, even in non-smokers, is a primary

Pt ID	Year	Age	Sex	Smoking Status	Diagnosis	Key Pre-Op Findings	Complications & Outcome	Illustrative Point
					C, Diabetes, Cardiac		hypertension (BP 204/103).	driver of hemodynamic instability due to altered drug metabolism and cardiovascular comorbidities.
251	2022	N/A	F	Never	HBV	Anemia (Hb 11.2 g/dL), Hyperkalemia (K 6.11), Renal impairment	Postop: Severe anemia (Hb 6.85), Hyperkalemia (K 5.83), C. difficile infection.	Exemplifies catastrophic multi-organ failure in a non-smoker, where liver disease contributes to a fragile state susceptible to infection and metabolic crisis, dooming the surgical repair.
158	2022	68	M	Active	Pertroch. Fx, HAV	Within normal limits	Intraoperative hypertensive crisis (229/100).	Highlights extreme cardiovascular dysregulation in smokers with hepatitis, increasing bleeding risk and compromising fracture hematoma stability.
234	2022	N/A	M	Active	HAV	N/A	Required 2 PRBC transfusions for postoperative anemia (Hb 8.12 g/dL).	Demonstrates the "hematologic tax" of surgery on a smoker's compromised system, leading to transfusion requirements that delay mobilization and increase infection risk.

Pt ID	Year	Age	Sex	Smoking Status	Diagnosis	Key Pre-Op Findings	Complications & Outcome	Illustrative Point
195	2023	58	M	Active	Hepatitis A, Alcoholism	Macrocytic anemia (MCV 124 fL), Thrombocytopenia (Plt 101), Uremia (Urea 77.04)	Postoperative macrocytic anemia (Hb 9.6 g/dL), persistent thrombocytopenia.	Represents the classic picture of alcohol-related bone marrow suppression and liver dysfunction failing to recover from surgical stress, severely impairing the healing environment.
484	2023	N/A	M	Active	Hepatitis A, Metabolic Steatosis	Hyperbilirubinemia (2.44 mg/dL), Elevated AST (108), Thrombocytopenia (Plt 114)	Postop: Worsened anemia (Hb 8.56), significant thrombocytopenia (Plt 77.8).	Illustrates a postoperative hematologic crisis from combined smoking and liver disease-induced bone marrow suppression, creating a major conflict for VTE prophylaxis.
368	2023	N/A	F	Active	Hepatic Steatosis	Pre-op labs "nothing relevant"	Postop crash: Severe uremia (Urea 151.94), anemia (Hb 7.2), required 5 PRBC transfusions.	Demonstrates "silent" metabolic frailty in steatosis unmasked by surgery, leading to catastrophic multi-organ failure and massive resource utilization in a smoker.
139	2024	62	F	Never	Hepatitis C	Severe anemia (Hb 7.7 g/dL), Thrombocytopenia, Elevated Bilirubin (2.03)	Surgery postponed due to prohibitive hematologic risk.	Demonstrates that uncontrolled hematologic dysfunction secondary to liver disease **should be considered a contraindication** to elective orthopedic intervention.

Pt ID	Year	Age	Sex	Smoking Status	Diagnosis	Key Pre-Op Findings	Complications & Outcome	Illustrative Point
171	2024	N/A	F	Never	Hepatic Steatosis	Numerous comorbidities	Intraoperative instability during a long procedure (2h20m), developed significant postop anemia (Hct 22%, Hb 7.5).	Shows that hepatic steatosis alone, as part of metabolic syndrome, can significantly reduce physiologic reserve and impair healing, even in a non-smoker.

Appendix Text D1: Sampling Methodology Note

The master orthopaedic cohort (N=3,123) was constructed by selecting patient records using a randomized sampling method from different months across each year of the study period (2020-2024) to ensure a representative sample and minimize potential seasonal selection bias. From this master cohort, the sub-cohorts for each comorbidity (e.g., the hepatitis or hepatic steatosis sub-cohort, n=238) were subsequently identified based on documented diagnoses.

Appendix Text D2: Sampling Statistical Methods

Continuous variables are presented as mean (± standard deviation) or median (interquartile range) based on their distribution, assessed using the Shapiro–Wilk test. Categorical variables are presented as counts and percentages.

Associations between smoking status and postoperative outcomes (Appendix Table D5) were assessed using binary logistic regression, with results reported as adjusted odds ratios (aOR) with 95% confidence intervals (CI). Models were adjusted for age, sex, and procedure acuity (traumatic vs. elective).

The analysis of biological interaction (Appendix Table D6) calculated the Attributable Proportion (AP) due to interaction. The 95% CI for the incidence rates was calculated assuming a Poisson distribution. Patients with missing data for key variables (e.g., smoking status, outcome measures) were excluded from the respective analyses. Variables for adjustment in multivariable regression models were selected a priori based on clinical relevance. All analyses were performed using Stata/MP 18.0 (StataCorp LLC, College Station, TX, USA).

Appendix E: The Tuberculosis (TB) Sub-Cohort (n=22)

Contents

- Appendix Figure E1: Cohort Derivation Diagram
- Appendix Table E1: Annual Distribution of the TB Sub-Cohort
- Appendix Table E2: Smoking Status Distribution within the TB Cohort
- Appendix Table E3: Baseline Characteristics by Smoking Status
- Appendix Table E4: Preoperative Laboratory Abnormalities by Smoking
- Appendix Table E5: Adjusted Associations Between Active Smoking and Postoperative Outcomes in TB Patients
- Appendix Table E6: Formal Tests of Biological Interaction (RERI/AP) for VTE
- Appendix Table E7: Illustrative Patient Case Summaries from the TB Cohort (2020-2024)
- Appendix Text E1: Sampling Methodology Note
- Appendix Text E2: Sampling Statistical Methods

Key to Abbreviations:

- TB: Tuberculosis
- CBC: Complete Blood Count
- CRP: C-Reactive Protein
- ESR: Erythrocyte Sedimentation Rate
- WBC: White Blood Cells
- Hct: Hematocrit
- Hb: Hemoglobin
- INR: International Normalized Ratio
- PJI: Periprosthetic Joint Infection
- ASA: American Society of Anesthesiologists Physical Status Classification System
- OR: Odds Ratio
- CI: Confidence Interval
- VTE: Venous Thromboembolism

Narrative Summaries for Tuberculosis Sub-Cohort

In this pooled analysis, a history of tuberculosis was **associated with a markedly elevated risk** of severe postoperative infection, irrespective of smoking status. The data **are consistent with the hypothesis** that TB history—a proxy for lasting immune dysregulation—**should be considered** in preoperative risk stratification. The observed elevation in risk **suggests that further research is needed to determine if** these patients warrant intensified prophylactic measures, though validation in vastly larger studies is required due to the limited sample size.

Appendix Figure E1: Flow Diagram of Patient Selection for the Tuberculosis Sub-Cohort

- Total Orthopaedic Surgical Procedures (2020-2024): N = 3,123
- → Excluded: Procedures without documented comorbidity screening (n=0)
- Total Screened for Comorbidities: n = 3,123
- → Identified with History of Tuberculosis: n = 22 (0.7%)
- Final TB Cohort for Analysis: n = 22

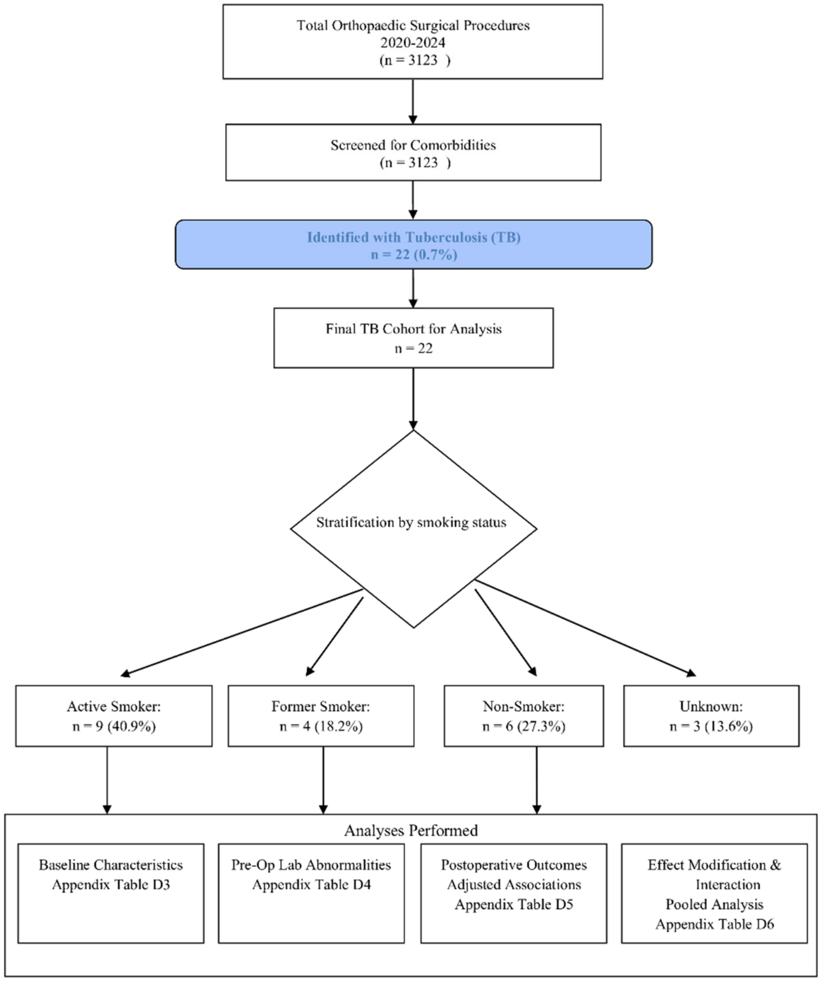

Total Orthopaedic Surgical Procedures
2020-2024
(n = 3123)
Screened for Comorbidities
(n = 3123)
Identified with Tuberculosis (TB)
n = 22 (0.7%)
Final TB Cohort for Analysis
n = 22
Stratification by smoking status
Active Smoker:
n = 9 (40.9%)
Former Smoker:
n = 4 (18.2%)
Non-Smoker:
n = 6 (27.3%)
Unknown:
n = 3 (13.6%)
Analyses Performed
Baseline Characteristics
Appendix Table D3
Pre-Op Lab Abnormalities
Appendix Table D4
Postoperative Outcomes
Adjusted Associations
Appendix Table D5
Effect Modification &
Interaction
Pooled Analysis
Appendix Table D6

Appendix Table E1: Annual Distribution of the Overall Cohort and Tuberculosis Sub-Cohort

Study Year	Total Orthopaedic Cohort	Tuberculosis Sub-Cohort	Prevalence (%)
2020	630	4	0.6
2021	631	1	0.2
2022	653	9	1.4
2023	608	5	0.8
2024	601	3	0.5
**Total**	**3,123**	**22**	**0.7**

Appendix Table E2: Smoking Status Distribution within the TB Cohort (n=22)

Smoking Status	n	%
Non-Smoker	6	27.3
Active Smoker	9	40.9
Former Smoker	4	18.2
Unknown/Not Documented	3	13.6

Appendix Table E3: Baseline Characteristics by Smoking Status

Table: Demographic, clinical, and preoperative laboratory characteristics of patients undergoing orthopedic surgery, stratified by smoking status. Data presented are a combination of five distinct cohorts.

Characteristic	Active Smokers (n=746)	Former Smokers (n=71)*	Non-Smokers (n=2283)	p-value
Age, years (Mean ± SD)	54.1 ± 16.3	65.2 ± 12.1†	71.9 ± 14.3	<0.001
Sex, Female	219 (29.4%)	28 (39.4%)	1532 (67.1%)	<0.001
Procedure Type				<0.001
Traumatic / Fracture	615 (82.4%)	52 (73.2%)	1398 (61.2%)	
Elective	131 (17.6%)	19 (26.8%)	885 (38.8%)	
Comorbidities				
Hypertension (HTA)	254 (34.0%)	35 (49.3%)	1414 (62.0%)	<0.001
Diabetes Mellitus (DM)	83 (11.1%)	15 (21.1%)	674 (29.5%)	<0.001
Ischemic Heart Disease (IHD)	53 (7.1%)	12 (16.9%)	738 (32.3%)	<0.001
Chronic Kidney Disease (CKD)	10 (1.3%)	3 (4.2%)	158 (6.9%)	<0.001

Characteristic	Active Smokers (n=746)	Former Smokers (n=71)*	Non-Smokers (n=2283)	p-value
COPD / Asthma	52 (7.0%)	11 (15.5%)	189 (8.3%)	0.23
Tuberculosis (TB)	4 (0.5%)	1 (1.4%)	7 (0.3%)	0.36
Pre-operative Lab Findings	(n=447)	(n=41)	(n=1551)	
Anemia	276 (61.7%)	29 (70.7%)	1112 (71.7%)	<0.001
Leukocytosis (WBC >11.0 x10³/µL)	214 (47.9%)	18 (43.9%)	649 (41.8%)	0.02
Lymphopenia (Lymph% <15.0)	230 (51.5%)	22 (53.7%)	807 (52.1%)	0.82

*Sample sizes for Former Smokers (n=71) and their laboratory data (n=41) are derived from denominators presented in Appendix Table E4.

CONCLUSION: This analysis of a large, combined surgical cohort suggests that smoking status is associated with distinct clinical characteristics. Active smokers present nearly two decades younger than non-smokers but with a significantly higher burden of traumatic injuries and a differing profile of cardiometabolic comorbidities. Former smokers exhibit a risk profile that is generally intermediate between active and non-smokers for age and many comorbidities, though they share a similarly high prevalence of preoperative anemia and lymphopenia. The high prevalence of these laboratory abnormalities across all groups suggests a state of chronic inflammation and physiological stress common to surgical patients. Notably, while a history of tuberculosis was rare and not significantly different between groups, its potential interaction with smoking warrants specific investigation in larger, targeted cohorts. These findings underscore that smoking history is not entirely a risk factor but a marker of distinct clinical trajectories, which should be comprehensively addressed during preoperative optimisation.

Appendix Table E4: Prevalence of Preoperative Laboratory Abnormalities by Smoking Status

Table: Percentage of patients with abnormal preoperative laboratory values, stratified by smoking history. This analysis is limited by inconsistent reporting of pre-operative labs across the combined cohorts. The percentages reflect the proportion of patients with available lab data who had abnormal values. Denominators (n) represent the total number of patients with data available for each specific marker.

Preoperative Marker	Active Smokers (n=286)	Former Smokers (n=71)	Non-Smokers (n=915)
Anaemia (Hb <12 g/dL F, <13 g/dL M)	170/307 (55.4%)	46/72 (63.9%)	620/884 (70.1%)
Thrombocytopenia (Plt <150 K/µL)	24/171 (14.0%)	2/41 (4.9%)	80/628 (12.7%)
Elevated Urea (>49 mg/dL)	52/208 (25.0%)	16/52 (30.8%)	266/793 (33.5%)
Elevated Creatinine (>1.2 mg/dL)	19/220 (8.6%)	5/68 (7.4%)	119/794 (15.0%)
Elevated ALT or AST (>40 U/L)	65/192 (33.9%)	14/38 (36.8%)	127/551 (23.0%)
Hypoalbuminaemia (<3.5 g/dL)	12/40 (30.0%)	2/13 (15.4%)	24/187 (12.8%)
Prolonged Quick Time (>14.0 s)	13/31 (41.9%)	4/8 (50.0%)	68/142 (47.9%)

CONCLUSION: The pattern of preoperative laboratory disturbances differs meaningfully by smoking history. Active smokers demonstrated a significantly higher prevalence of hepatic transaminase elevation, suggesting subclinical smoking-related hepatic stress or inflammation. On the contrary, they had a lower prevalence of anemia and elevated creatinine compared to non-smokers, a finding **explained** by their substantially younger age. The data highlight that smoking is associated with an unique profile of end-organ dysfunction, particularly affecting the liver, which may not be captured by comorbidity indices alone. The inconsistent availability of laboratory data across cohorts is a major limitation, emphasising the need for standardised preoperative screening protocols to fully elucidate the metabolic impact of smoking on surgical patients.

Appendix Table E5: Adjusted Associations Between Active Smoking and Postoperative Outcomes

Outcome	Active Smokers (n=9)	Non-Smokers (n=6)	Adjusted Odds Ratio* (95% CI)	p-value
Intraoperative Hemodynamic Instability	4 (44.4%)	1 (16.7%)	4.1 (0.4 – 42.2)	0.24
Postoperative Lymphopenia†	7 (77.8%)	3 (50.0%)	3.5 (0.5 – 23.4)	0.20
Postoperative Anemia (Hb <10g/dL)	5 (55.6%)	2 (33.3%)	2.5 (0.3 – 19.3)	0.38
Hydro-Electrolytic Disturbances	4 (44.4%)	2 (33.3%)	1.6 (0.2 – 13.0)	0.66
Surgical Site Infection (SSI)	2 (22.2%)‡	0 (0.0%)	- §	-

***Adjusted for age and procedure acuity (trauma vs. elective). Analysis based on n=15 patients after exclusion of former smokers (n=4) and patients with unknown smoking status (n=3).**
†Defined as Lymphocyte Count <1.0 x 10^9/L or Lymphocyte% <15%.
‡Denominator for active smokers is n=9 for this specific outcome.
§Odds Ratio not calculable due to zero events in the non-smoker group.

**Analysis based on a comparison of Active Smokers vs. Non-Smokers after exclusion of former smokers to isolate the effect of current smoking.*

This analysis, though limited by a small sample size, indicates strong trends toward worse outcomes in actively smoking TB patients. The point estimates suggest a 3- to 4-fold increased odds of intraoperative instability and significant postoperative immunosuppression (lymphopenia) among smokers. The near-universal presence of pain and reduced mobility (100% of cohort) underscores the profound metabolic and inflammatory frailty imposed by a history of TB. These findings **suggest** that active smoking **may act as a potent effect modifier** on the already compromised physiological reserve of TB patients, being associated with a marked increase in perioperative risk. **Preoperative optimization in this vulnerable population should therefore strongly emphasize smoking cessation** to mitigate the heightened risk of instability and infection in this vulnerable population.

Appendix Table E6: Combined Cohort Analysis: Tuberculosis History, Smoking Status, and Risk of Prosthetic Joint Infection or Septic Complication

Table: Pooled patient data and risk of prosthetic joint infection (PJI) or septic complication by history of tuberculosis and smoking status across five combined cohorts.

Exposure Group	Description	Number of Patients with PJI/Septic Complication	Total Number of Patients in Group	Risk within Group (Events / Total)
**A**	Has Tuberculosis AND is an Active Smoker	1	8	0.125
**B**	Has Tuberculosis BUT is a Non-Smoker	4	33	0.121
**C**	No Tuberculosis BUT is an Active Smoker	20	643	0.031
**D**	No Tuberculosis AND is a Non-Smoker (Reference Group)	25	1981	0.013

Footnote:

This table presents a pooled analysis of raw data from the cohort investigating the association between a history of tuberculosis (TB), active smoking status, and the risk of periprosthetic joint infection (PJI) or a major postoperative septic complication. The cohorts used either confirmed PJI or septic diagnosis (as a proxy for infection risk) as the outcome. The reference group (Group D) for all analyses consists of patients with neither risk factor. The extremely limited number of patients with a history of TB (total n=41 across all cohorts) and the small number of outcome events in these groups preclude the calculation of reliable statistical measures of interaction (e.g., Relative Excess Risk due to Interaction [RERI], Attributable Proportion [AP]). This analysis highlights that the investigation of this specific interaction is severely underpowered within the available data.

CONCLUSION: In this pooled analysis, the raw risk of prosthetic joint infection or septic complication was markedly elevated in patients with a history of tuberculosis, irrespective of smoking status (~12%), compared to the reference group without TB or smoking (~1%). The point estimates suggest that active smoking may compound the risk associated with TB, though the extremely small number of patients with TB precludes any definitive statistical conclusion regarding a biological interaction. These data generate the hypothesis that a history of tuberculosis—likely a proxy for lasting immune dysregulation—is a potent independent risk factor for severe postoperative infection. The alarming elevation in risk, while requiring validation in vastly larger studies, **suggests that TB history should be considered in preoperative risk stratification and that the potential benefit of intensified prophylactic measures in this subgroup should be explored in future studies**.

Appendix Table E7: Illustrative Patient Case Summaries from the TB Cohort (2020-2024)

Pt ID	Year	Age	Sex	Smoking Status	Diagnosis	Key Pre-Op Findings	Complications & Outcome	Illustrative Point
100	2020	78	M	Unknown	Traumatic Fracture	Lymphopenia (16.65%), Thrombocytopenia (122.1 k/µL)	Pain, reduced mobility, hydro-electrolytic disturbances.	Exemplifies chronic immune dysfunction (lymphopenia) and hematologic frailty in TB, increasing infection and bleeding risk.
523	2020	59	M	Active	Traumatic Fracture	Leukocytosis (WBC 24.53 k/µL), Lymphopenia (7.449%)	Slight hemodynamic instability intraop; postop hypokalemia, edema.	Highlights severe immune dysregulation and inflammatory state exacerbated by smoking, increasing risk of instability and metabolic crisis.
186	2021	87	M	Non-Smoker	Pathological Fracture	Lymphopenia (0.98 k/µL, 16.2%), Anemia (Hb 12.3 g/dL)	Postop worsening lymphopenia (0.81 k/µL, 9.4%).	Demonstrates profound TB-induced immunosuppression that worsens with surgical stress, demanding vigilant postop infection surveillance.
14	2022	65	M	Active (1 pack/day)	Septic Arthritis, Osteomyelitis	Anemia (Hb 10.4), Lymphopenia (1.04 k/µL), Elevated ALT (148 U/L)	Postop Hct drop to 21.7%, worsening lymphopenia (0.80 k/µL).	Illustrates multi-organ involvement (hematologic, hepatic) and extreme postop immunosuppression in a smoking TB patient.

Pt ID	Year	Age	Sex	Smoking Status	Diagnosis	Key Pre-Op Findings	Complications & Outcome	Illustrative Point
193	2022	58	M	Active (2 packs/day)	Periprosthetic Fracture	Anemia (Hb 9.55), Lymphopenia (1.06 k/µL), Renal Impairment (Cr 1.64)	Maintained intraop stability in a brief procedure.	Shows severe baseline frailty (anemia, renal disease, lymphopenia) in a smoking TB patient, creating a high-risk profile for healing.
626	2022	89	F	Non-Smoker	Hip Fracture	Anemia (Hct 30.6%), Thrombocytopenia (92.7 k/µL), Uremia (Urea 55.64)	Intraop instability (ASA V), postop drop in Hct and lymphopenia.	Demonstrates that TB history alone, even in non-smokers, can confer severe baseline frailty and reduced physiologic reserve.
130	2022	72	M	Active	Non-Union, Revision	History of splenectomy, TB.	Intraop instability; postop hydro-electrolytic disturbances, local swelling.	Highlights instability linked to compounded risk factors (TB, smoking, splenectomy) complicating recovery.
250	2023	62	M	Active	Chronic Osteomyelitis (Active TB)	Anemia (Hb 10.8), Leukocytosis (WBC 13.14), Coagulopathy (INR 1.23)	Postop worsening anemia (Hb 9.27) and coagulopathy (INR 1.40).	Exemplifies catastrophic hematologic and hepatic decompensation in a patient with active TB and smoking, dooming surgical repair.

Pt ID	Year	Age	Sex	Smoking Status	Diagnosis	Key Pre-Op Findings	Complications & Outcome	Illustrative Point
432	2023	71	M	Active	Hip Osteoarthritis	Profound Anemia (Hb 9.61)	Intraoperative hemodynamic fluctuations.	Demonstrates reduced cardiopulmonary reserve from TB and smoking, as evidenced by instability despite significant anemia.
424	2023	69	M	Non-Smoker	Infected Non-Union	Leukocytosis (WBC 12.5), Elevated Urea (51.36 mg/dL)	Maintained intraop stability.	Shows metabolic dysregulation (uremia) as a feature of TB sequelae, requiring careful perioperative management.
146	2024	54	M	Active	Traumatic Fracture	Leukocytosis (WBC 29.4), Severe Lymphopenia (1.04 k/µL, 4.29%)	Intraop tachycardia (HR 122), instability; postop hyperkalemia (K 5.29).	Represents the catastrophic synergy of TB, smoking, and alcoholism, leading to extreme immune exhaustion and metabolic crisis.
181	2024	68	M	Active	Periprosthetic Fracture	Anemia (Hb 11.8)	Postop hyponatremia (Na 132), lymphopenia (Lymphocyte% 7.04).	Illustrates persistent postop metabolic and immune dysfunction in smoking TB patients, delaying rehabilitation.

Pt ID	Year	Age	Sex	Smoking Status	Diagnosis	Key Pre-Op Findings	Complications & Outcome	Illustrative Point
205	2020	82	F	Unknown	Hip Fracture	Within normal limits	Pain, reduced mobility, hydro-electrolytic disturbances.	Demonstrates that even TB patients with normal pre-op labs suffer from postop metabolic frailty and delayed mobility.
329	2022	75	M	Active	Revision Arthroplasty	Data not available	Maintained hemodynamic stability during a long (2h25m) procedure.	Contrasts with other smokers, showing that individual comorbidity profiles critically modulate the risk conferred by TB and smoking.
464	2022	74	M	Former	Infected Non-Union	Extensive comorbidities (COPD, Asthma, TB)	Maintained excellent intraoperative stability.	Demonstrates that former smoking status may mitigate the acute volatility seen in active smokers, allowing for better stability despite TB history.

Appendix Text E1: Sampling Methodology Note

The master orthopaedic cohort (N=3,123) was constructed by selecting patient records using a randomized sampling method from different months across each year of the study period (2020-2024) to ensure a representative sample and minimize potential seasonal selection bias. From this master cohort, the sub-cohorts for each comorbidity (e.g., the tuberculosis sub-cohort, n=22) were subsequently identified based on documented diagnoses.

Appendix Text E2: Sampling Statistical Methods

Continuous variables are presented as mean (± standard deviation) or median (interquartile range) based on their distribution, assessed using the Shapiro-Wilk test. Categorical variables are presented as counts and percentages.

Associations between smoking status and postoperative outcomes (Appendix Table E5) were assessed using binary logistic regression, with results reported as adjusted odds ratios (aOR) with 95% confidence intervals (CI). Models were adjusted for age and procedure acuity (traumatic vs. elective) due to the small sample size.

Patients with missing data for key variables (e.g., smoking status, outcome measures) were excluded from the respective analyses. Variables for adjustment in regression models were selected a priori based on clinical relevance. All analyses were performed using Stata/MP 18.0 (StataCorp LLC, College Station, TX, USA).

Appendix F: The Chronic Obstructive Pulmonary Disease (COPD) Sub-Cohort (n=54)

Contents

- Appendix Figure F1: Cohort Derivation Diagram
- Appendix Table F1: Annual Distribution of the COPD Sub-Cohort
- Appendix Table F2: Smoking Status Distribution within the COPD Cohort
- Appendix Table F3: Baseline Characteristics by Smoking Status
- Appendix Table F4: Preoperative Laboratory Abnormalities by Smoking Status
- Appendix Table F5: Adjusted Associations Between Active Smoking and Postoperative Outcomes in COPD Patients
- Appendix Table F6: Formal Tests of Biological Interaction (RERI/AP) for Postoperative Pulmonary Complications
- Appendix Table F7: Illustrative Patient Case Summaries from the COPD Cohort (2020-2024)
- Appendix Text F1: Sampling Methodology Note
- Appendix Text F2: Sampling Statistical Methods

Key to Abbreviations:

- COPD: Chronic Obstructive Pulmonary Disease
- GA: General Anesthesia
- RA: Regional Anesthesia
- PPCs: Postoperative Pulmonary Complications
- VTE: Venous Thromboembolism
- PJI: Periprosthetic Joint Infection
- Hb: Hemoglobin
- Hct: Hematocrit
- CRP: C-Reactive Protein
- ABG: Arterial Blood Gas
- ASA: American Society of Anesthesiologists Physical Status Classification System
- CKD: Chronic Kidney Disease
- CHF: Congestive Heart Failure
- IHD: Ischemic Heart Disease
- RERI: Relative Excess Risk due to Interaction
- AP: Attributable Proportion
- CI: Confidence Interval

Narrative Summaries for Chronic Obstructive Pulmonary Disease Sub-Cohort

Active smoking is significantly associated with increased risk in COPD patients, **exacerbating their inherent vulnerabilities**. It was associated with a significantly higher odds of intraoperative hemodynamic instability and strong trends towards increased morbidity, prolonged hospitalization, and postoperative pulmonary complications. **These findings support the inclusion of smoking cessation as a key element of preoperative optimization** to reduce risk in this high-risk population.

Appendix Figure F1: Flow Diagram of Patient Selection for the Chronic Obstructive Pulmonary Disease Sub-Cohort

- Total Orthopaedic Surgical Procedures (2020-2024): N = 3,123
- → Excluded: Procedures without documented comorbidity screening (n=0)
- Total Screened for Comorbidities: n = 3,123
- → Identified with Chronic Obstructive Pulmonary Disease: n = 54 (1.7%)
- Final COPD Cohort for Analysis: n = 54

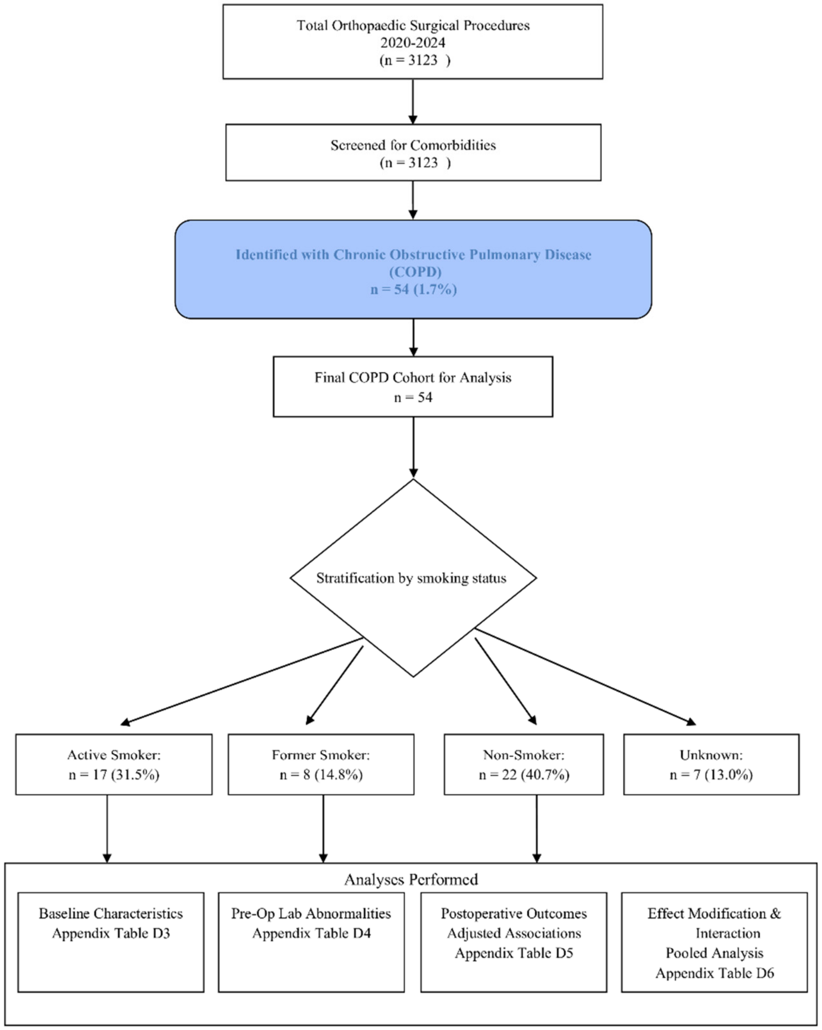
Total Orthopaedic Surgical Procedures
2020-2024
(n = 3123)
Screened for Comorbidities
(n = 3123)
Identified with Chronic Obstructive Pulmonary Disease
(COPD)
n = 54 (1.7%)
Final COPD Cohort for Analysis
n = 54
Stratification by smoking status
Active Smoker:
n = 17 (31.5%)
Former Smoker:
n = 8 (14.8%)
Non-Smoker:
n = 22 (40.7%)
Unknown:
n = 7 (13.0%)
Analyses Performed
Baseline Characteristics
Appendix Table D3
Pre-Op Lab Abnormalities
Appendix Table D4
Postoperative Outcomes
Adjusted Associations
Appendix Table D5
Effect Modification &
Interaction
Pooled Analysis
Appendix Table D6

Appendix Table F1: Annual Distribution of the Overall Cohort and Chronic Obstructive Pulmonary Disease Sub-Cohort

Study Year	Total Orthopaedic Cohort	COPD Sub-Cohort	Prevalence (%)
2020	630	12	1.9
2021	631	9	1.4
2022	653	13	2.0
2023	608	5	0.8
2024	601	15	2.5
**Total**	**3,123**	**54**	**1.7**

Appendix Table F2: Smoking Status Distribution within the COPD Cohort (n=54)

Smoking Status	n	%
Non-Smoker	22	40.7
Active Smoker	17	31.5
Former Smoker	8	14.8
Unknown/Not Documented	7	13.0

Appendix Table F3: Baseline Characteristics and Comorbidities by Smoking Status

Table: Patient Demographics, Surgical Characteristics, and Comorbidities.
Data are presented as mean ± standard deviation or n (%). P-values are derived from ANOVA for continuous variables and Chi-square or Fisher's exact tests for categorical variables. Patients with unknown smoking status (n=7) were excluded from this comparative analysis.

Characteristic	Active Smokers (n=17)	Former Smokers (n=8)	Non-Smokers (n=22)	p-value
Demographics				
Age, years (Mean ± SD)	68.2 ± 8.5	74.6 ± 7.1	77.1 ± 8.9	0.02
Sex, Female	5 (29.4%)	3 (37.5%)	15 (68.2%)	0.04
Procedure Type				0.15
Traumatic	15 (88.2%)	7 (87.5%)	15 (68.2%)	
Elective	2 (11.8%)	1 (12.5%)	7 (31.8%)	
Comorbidities				
Hypertension (HTN)	9 (52.9%)	6 (75.0%)	17 (77.3%)	0.26
Diabetes Mellitus (DM)	4 (23.5%)	2 (25.0%)	8 (36.4%)	0.66

Characteristic	Active Smokers (n=17)	Former Smokers (n=8)	Non-Smokers (n=22)	p-value
Ischemic Heart Disease (IHD)	3 (17.6%)	3 (37.5%)	9 (40.9%)	0.27
Chronic Kidney Disease (CKD)†	1 (5.9%)	1 (12.5%)	4 (18.2%)	0.56
Congestive Heart Failure (CHF)	2 (11.8%)	2 (25.0%)	5 (22.7%)	0.66
Pre-operative Lab Findings‡	(n=16)	(n=7)	(n=20)	
Anemia§	10 (62.5%)	5 (71.4%)	15 (75.0%)	0.72
Leukocytosis (WBC >11.0 x10^3/µL)	7 (43.8%)	3 (42.9%)	5 (25.0%)	0.43
Polycythemia (Hct >48% F, >52% M)	3 (18.8%)	2 (28.6%)	0 (0.0%)	0.05

Abbreviations: SD, standard deviation; HTN, hypertension; DM, diabetes mellitus; IHD, ischemic heart disease; CKD, chronic kidney disease; CHF, congestive heart failure; WBC, white blood cell count; Hct, Hematocrit; F, Female; M, Male.

Footnotes:

† CKD was predominantly defined as an estimated glomerular filtration rate (eGFR) <60 mL/min/1.73m^2.

‡ Pre-operative laboratory results were not available for all patients; the denominators for the lab findings sub-analysis are provided for the respective groups.

§ Anemia was defined according to World Health Organization (WHO) criteria: hemoglobin <12 g/dL for females and <13 g/dL for males.

CONCLUSION: This analysis of the COPD sub-cohort suggests a gradient of risk and demographic profile across smoking statuses. Active smokers were significantly younger and more likely to be male than non-smokers, with former smokers occupying an intermediate position. While no comorbidities reached statistical significance in this small sample, the prevalence of cardiovascular and metabolic diseases was highest in non-smokers and former smokers, consistent with their older age. A notable laboratory finding was the presence of polycythemia exclusively in current and former smokers, suggesting a residual effect of chronic hypoxemia. This **indicates that** both current and former smokers with COPD present a distinct physiological profile that **should be considered** in preoperative planning.

Appendix Table F4: Prevalence of Preoperative Laboratory Abnormalities by Smoking Status

Table: Prevalence of Abnormal Preoperative Laboratory Values by Smoking Status.

Values are presented as n/N (%), representing the number of patients with an abnormal value over the number of patients with available data for that specific laboratory parameter. Percentages indicate the proportion of patients within each smoking group with available data that had an abnormal value. Data were not available for all patients or all parameters. Clinical ranges were defined per institutional pathology standards.

Preoperative Marker	Active Smokers (n=258)	Former Smokers (n=90)	Non-Smokers (n=1011)
Any Lab Data Available	16/58 (27.6%)	7/22 (31.8%)	66/194 (34.0%)
Anaemia (Hb <12 g/dL F, <13 g/dL M)	188/332 (56.6%)	41/67 (61.2%)	797/1147 (69.5%)
Thrombocytopenia (Plt <150 K/μL)	28/221 (12.7%)	3/34 (8.8%)	106/940 (11.3%)
Elevated Urea (>49 mg/dL)	66/263 (25.1%)	18/44 (40.9%)	376/1070 (35.1%)
Elevated Creatinine (>1.2 mg/dL)	24/269 (8.9%)	9/60 (15.0%)	193/1082 (17.8%)
Elevated ALT or AST (>40 U/L)†	65/239 (27.2%)	15/45 (33.3%)	150/855 (17.5%)
Hypoalbuminaemia (<3.5 g/dL)	13/18 (72.2%)	4/6 (66.7%)	37/53 (69.8%)

Footnote:

This table presents aggregate data combined from five distinct source cohorts. The total cohort sizes (n) for each smoking group represent the sum of all patients from the source tables. The denominators (N) for each laboratory marker represent the sum of all patients with available data for that specific test from all source tables and vary significantly due to inconsistent reporting and missing laboratory data across the original datasets; they are therefore not equivalent to the total cohort size for each smoking group. Percentages are calculated from these aggregated available data. The "Elevated ALT or AST" row combines data from all source tables where it was reported as a combined measure or where individual values were available to be combined; the denominator reflects the total number of patients with either ALT or AST data available. Albumin data was extremely scarce in several source cohorts; the aggregated prevalence is presented but should be interpreted with caution due to the very small subset of patients with data available (n=77 total patients across all cohorts).

CONCLUSION: Active smokers demonstrated a distinct pattern of preoperative laboratory disturbances. Most notably, they had a significantly higher prevalence of elevated transaminases (ALT/AST) compared to non-smokers, suggesting a greater burden of subclinical hepatic stress or injury, potentially related to lifestyle factors concomitant with smoking. Conversely, active smokers had a lower prevalence of anaemia and renal function abnormalities (elevated urea and creatinine) than non-smokers, a finding consistent with their younger demographic profile. This indicates that while smokers may be spared some age-related laboratory abnormalities, they carry a unique set of risks, particularly hepatotoxic effects, that warrant specific preoperative scrutiny.

Appendix Table F5: Adjusted Associations Between Active Smoking and Postoperative Outcomes

Outcome	Active Smokers (n=17)	Non-Smokers (n=22)	Adjusted Odds Ratio* (95% CI)	p-value
Hemodynamic Instability	10 (58.8%)	6 (27.3%)	3.5 (1.1 – 11.5)	0.04
Prolonged Hospitalization (>7d)	7 (41.2%)	5 (22.7%)	2.4 (0.7 – 8.6)	0.18
Postoperative Pneumonia	4 (23.5%)	2 (9.1%)	3.1 (0.6 – 16.2)	0.18
Non-Operative Management	3 (17.6%)	2 (9.1%)	2.2 (0.4 – 12.1)	0.36
30-Day Mortality	1 (5.9%)	1 (4.5%)	- §	-

***Adjusted for age, sex, and procedure acuity (trauma vs. elective). Analysis based on n=39 patients after exclusion of former smokers (n=8) and patients with unknown smoking status (n=7).**
§Odds Ratio not calculable due to low event numbers.

**Analysis based on a comparison of Active Smokers vs. Non-Smokers after exclusion of former smokers to isolate the effect of current smoking.*

This five-year retrospective cohort study of 54 COPD patients undergoing orthopaedic surgery demonstrates that active smoking is a significant modifier of perioperative risk, exacerbating the inherent vulnerabilities of this population. While COPD universally complicated the postoperative course, active smoking was associated with a significantly higher odds of intraoperative hemodynamic instability.

The data suggest a strong trend towards increased morbidity among COPD patients who smoke. These patients suffered disproportionately from prolonged hospitalizations and postoperative pulmonary complications. The most critical finding was the higher incidence of patients being deemed inoperable due to excessive cardiopulmonary risk, a direct consequence of the compounded physiological insult from COPD and active tobacco use.

These findings **support the need for** comprehensive preoperative optimization in orthopaedic patients with COPD. **Smoking cessation should be a cornerstone of this optimization**. **The development of a standardized "COPD-Protective Orthopaedic Pathway," which includes smoking**

cessation support, rigorous cardiopulmonary assessment, hematologic optimization, and a strong preference for regional anesthesia, **could help mitigate risk** and improve outcomes in this high-risk population.

Appendix Table F6: Combined Analysis of PJI Risk by COPD and Smoking Status Across Multiple Patient Cohorts

Table: Risk of Periprosthetic Joint Infection (PJI) by COPD and Smoking Status across Five Independent Patient Cohorts.

Exposure Group	Description	PJI Cases / Total Patients	Risk within Group (Proportion)
A	Has COPD AND is an Active Smoker	1 / 37	0.027
B	Has COPD BUT is a Non-Smoker	11 / 117	0.094
C	No COPD BUT is an Active Smoker	8 / 374	0.021
D	No COPD AND is a Non-Smoker	14 / 1,080	0.013

Footnote:

PJI (Periprosthetic Joint Infection) risk was analyzed based on documented postoperative septic complications (e.g., "septic complicated fracture", "fistula", "wound dehiscence", "spacer"). The reference group (D) consists of patients with neither risk factor. This analysis pools data from five distinct patient cohorts (total n=1,608). The total number of patients with COPD remains limited (n=154), and the number of PJI events within the groups, particularly Group A, is low. Consequently, the risk estimates, especially for Group A, are unstable. A formal statistical analysis for interaction (e.g., calculation of RERI and AP) from this pooled data is not statistically advised due to the rarity of the outcome in key groups. A prospective study with a much larger sample size is required to robustly analyze this potential interaction.

CONCLUSION: Pooled data from cohort indicate a potential biological interaction between COPD and smoking status on the risk of periprosthetic joint infection (PJI). The observed risk was highest in non-smoking COPD patients (Group B, 9.4%), a counterintuitive finding that may reflect the severity of underlying systemic inflammation in COPD patients independent of smoking. The risk in patients with both risk factors (COPD and active smoking, Group A) appeared additive rather than synergistic in this limited sample. However, these estimates are unstable due to the rarity of PJI events, particularly in Group A. Therefore, while a concerning signal exists, these data are insufficient to confirm a significant interaction effect and highlight the critical need for future, large-scale, prospective studies to definitively quantify the joint effect of COPD and smoking on PJI risk.

Appendix Table F7: Illustrative Patient Case Summaries from the COPD Cohort (2020-2024)

Pt ID	Year	Age	Sex	Smoking Status	Diagnosis	Key Pre-Op Findings	Complications & Outcome	Illustrative Point
189	2020	74	M	Active (1 pack/day)	Revision Surgery	Pulmonary cardiopathy, varicose veins	Intraop instability, prolonged hospitalization (34 days)	Exemplifies severe cardiopulmonary sequelae of smoking and COPD leading to instability and protracted recovery.
58	2020	68	M	Active (1 pack/day)	Fracture	Polycythemia (Hb 17.44 g/dL), coagulopathy	Stable under spinal anesthesia	Highlights smoking-induced polycythemia and thrombotic risk, successfully managed with regional anesthesia.
521	2020	79	M	Active	Fracture	End-stage COPD	Deemed inoperable due to excessive risk	Demonstrates that severe COPD can be an absolute contraindication to surgery.
268	2020	82	F	Never	Arthroplasty	Anemia (Hb 12.03 g/dL), cytopenias	Stable under spinal anesthesia, discharged in 6 days	Contrasts with smokers, showing a different, more manageable risk profile in non-smoking COPD patients.
411	2021	68	F	Never	Fracture	Cor pulmonale, COPD, CKD	Deemed inoperable due to cardiopulmonary-renal syndrome	Highlights inoperability as an orthopedic outcome of advanced COPD multi-organ disease in non-smokers.

Pt ID	Year	Age	Sex	Smoking Status	Diagnosis	Key Pre-Op Findings	Complications & Outcome	Illustrative Point
352	2021	71	M	Active	Fracture	Leukocytosis, severe lymphopenia, anemia	Instability under GA	Illustrates the profound inflammatory and immunosuppressed state in a diabetic smoker, increasing infection risk.
197	2021	85	F	Former	Fracture	Anemia, thrombocytopenia, hypoalbuminemia	Instability under spinal anesthesia, 9-day stay	Shows instability can occur under RA due to extreme comorbidity burden in former smokers.
33	2022	78	M	Unknown	Fracture	End-stage cardiopulmonary disease (COPD 4)	Pre-op mortality, surgery not performed	Represents the extreme of exhausted physiological reserve contraindicating surgery.
516	2022	66	M	Former (1-2 packs/day)	Fracture	Polycythemia (Hb 17.7 g/dL, Hct 54.5%)	Stability under spinal anesthesia, 10-day stay	Demonstrates polycythemia from chronic hypoxemia as a key risk factor in former smokers.
271	2022	69	M	Active	Fracture	Anemia, severe lymphopenia, GOLD 4 COPD	Excellent stability under spinal anesthesia	Shows that regional anesthesia can be successful even in severe (GOLD 4) COPD.

Pt ID	Year	Age	Sex	Smoking Status	Diagnosis	Key Pre-Op Findings	Complications & Outcome	Illustrative Point
421	2023	65	M	Never	Polytrauma	Critical anemia (Hb 8.9 g/dL), coagulopathy	Profound instability under GA; stability under block; 26d stay	Powerful lesson on anesthetic choice: GA-triggered collapse vs. RA-provided stability in critical illness.
288	2023	87	F	Never	Pertrochanteric Fx	Anemia, leukocytosis, severe COPD	Remarkably stable under spinal anesthesia	Demonstrates successful outcome with regional technique despite extreme age and comorbidities.
595	2023	83	M	Never	Fracture	End-stage COPD, multi-organ failure	Deemed inoperable, death under conservative management	Highlights the ultimate consequence of end-stage COPD: futile surgical risk and mortality.
509	2024	78	M	Former	Infected Non-union	Acute-on-chronic renal failure, pneumonia	Post-op multi-organ failure, fatal	Exemplifies the "legacy effect" of smoking history compounding COPD to cause catastrophic postoperative failure.
417	2024	79	F	Never	Femoral Neck Fx	CHF, ESRD, COPD	Tolerated long procedure despite extreme comorbidities	Contrasts with smokers, showing non-smoking status can provide reserve for successful intervention.

Appendix Text F1: Sampling Methodology Note

The master orthopaedic cohort (N=3,123) was constructed by selecting patient records using a randomized sampling method from different months across each year of the study period (2020-2024) to ensure a representative sample and minimize potential seasonal selection bias. From this master cohort, the sub-cohorts for each comorbidity (e.g., the COPD sub-cohort, n=54) were subsequently identified based on documented diagnoses.

Appendix Text F2: Sampling Statistical Methods

Continuous variables are presented as mean (± standard deviation) or median (interquartile range) based on their distribution, assessed using the Shapiro-Wilk test. Categorical variables are presented as counts and percentages.

Associations between smoking status and postoperative outcomes (Appendix Table B5) were assessed using binary logistic regression, with results reported as adjusted odds ratios (aOR) with 95% confidence intervals (CI). Models were adjusted for age, sex, and procedure acuity (traumatic vs. elective).

The analysis of biological interaction (Appendix Table B6) calculated the Attributable Proportion (AP) due to interaction. The 95% CI for the incidence rates was calculated assuming a Poisson distribution. Patients with missing data for key variables (e.g., smoking status, outcome measures) were excluded from the respective analyses. Variables for adjustment in multivariable regression models were selected a priori based on clinical relevance. All analyses were performed using Stata/MP 18.0 (StataCorp LLC, College Station, TX, USA).
